# An analysis of enrollment rates by admitting service for a secondary analysis of enrollment data from a randomized smoking-cessation trial (FIESTA)

**DOI:** 10.1371/journal.pone.0345267

**Published:** 2026-11-04

**Authors:** Kayla Koffler, Michelle He, Joseph A. Ladapo, Scott Sherman

**Affiliations:** 1 School of Medicine, New York Medical College, Valhalla, New York, United States of America; 2 Department of Population Health, NYU Grossman School of Medicine, New York, New York, United States of America; 3 Division of General Internal Medicine, University of Florida College of Medicine, Gainesville, United States of America; Yale University, UNITED STATES OF AMERICA

## Abstract

**Introduction:**

Enrollment into clinical trials is challenging and has a major effect on the feasibility and the generalizability of a study. We used data from a previously completed hospital-based smoking cessation study to determine if enrollment rates varied by hospital admitting service, specifically comparing Medicine and Psychiatry departments.

**Methods:**

We examined enrollment in the Financial Incentives for Smoking Cessation (FIESTA) study, which was conducted at the Veterans Affairs New York Harbor Healthcare System. FIESTA tested the effectiveness of financial incentives on smoking cessation among hospitalized patients. A total of 268 patients were eligible, with 182 enrolled in the original FIESTA study. In our secondary analysis, a LASSO approach was applied to the data set to create a model examining admitting service, age, sex, race and ethnicity, education, and number of cigarettes per day and their association with enrollment.

**Results:**

The enrollment rates were higher for patients admitted to the Psychiatry service (80.2%) than to the Medicine and other services (59.9%). Patients with a high school education or less also had a higher enrollment rate (87.2%) than those with higher education (57.7%). Increasing age was associated with lower odds of enrollment (aOR = 0.97, 95% CI: 0.94, 0.99). Hispanic ethnicity appeared to associate with a higher odds of enrollment compared to non-Hispanic White (aOR = 2.52, 95% CI 1.11, 5.75). Higher odds of enrollment were observed among patients admitted to the Psychiatry service (aOR = 2.51, 95% CI: 1.23–5.12), while lower odds were observed among those with higher education (aOR = 0.17, 95% CI: 0.06–0.49).

**Conclusions:**

This research suggests that smoking cessation programs can effectively enroll a diverse patient population, including typically underrepresented groups such as those with lower education status and those with mental health diagnoses. Such enrollment efforts would enable studies to be more generalizable to the smoking population. Findings also emphasize the potential of urban hospital settings to support equitable enrollment efforts. The present work is a secondary analysis of the FIESTA RCT (NCT02506829). Future research should explore additional demographic factors, such as comorbidities and confidence in quitting, to better understand motivations for participation.

## Introduction

While smoking rates have declined from 20.9% in 2005 to 11.5% in 2023, approximately 28.3 million adults still smoked in 2021 in the United States. Further, 16 million Americans are living with an illness related to smoking [1]. Smoking cessation rates have not been uniform across populations. Many groups smoke at rates two to three times that of the general population, including people with low-income, people with mental health diagnoses, and other important groups. Tobacco control interventions are often targeted to these populations with higher rates of smoking [2].

Delivering an effective tobacco control intervention within the context of a pragmatic trial requires enrolling participants and retaining them in the study. Imbalances in enrollment could further perpetuate disparities in smoking rates. Previous research has determined that key factors for successful patient enrollment are clear communication, patient and provider motivation, patient convenience, and positive patient attitudes [3,4]. For smoking cessation in particular, motivation poses a unique challenge to enrollment compared to studies involving conditions or behaviors with more universal motivation for change.

Hospitalization is a window of opportunity to motivate people to quit smoking, and many hospital-based programs have been developed and implemented. One recent study in Turkey showcased that hospitalized patients approached for smoking cessation achieved a quit rate of nearly half [5]. For successful enrollment in the hospital, existing structures facilitating patient-provider relationships must be considered. Taking into account departmental differences and types of patient comorbidities may be useful when strategizing enrollment methods.

We used data from a previous pragmatic clinical trial to examine whether enrollment rates varied by admitting service. The Financial Incentives for Smoking Cessation (FIESTA) study was a randomized control trial (NCT 02506829) evaluating a financial incentive intervention for smoking cessation for hospitalized patients [6]. Our primary objective was to determine whether rates of FIESTA participant enrollment were higher on the Medical Service than on the Psychiatry Service. This work is a secondary analysis of enrollment data from the FIESTA trial (NCT 02506829). Given the exploratory nature of this study, analyses comparing enrollment outcomes across services were intended to generate preliminary insights rather than formally test prespecified hypotheses.

## Methods

### Study design

In FIESTA, patients hospitalized at the Manhattan campus of the Veterans Affairs (VA) New York Harbor Healthcare System, part of the Veterans Health Administration between July 15, 2015, to March 27, 2018. Of these, 268 were eligible and 182 were enrolled in the FIESTA trial and subsequently randomized. Participants who were enrolled in the study were randomly assigned to either enhanced usual care or to receive financial incentives in addition to enhanced care. Randomization was conducted with a computer-generated block randomization design with an allocation ratio of 1:1. Participants and study staff were not blinded to treatment assignment. The randomization procedure is not relevant to this study as we are solely commenting on the enrollment process. In the incentive arm, participants could receive up to $550 if they met all targets for treatment (e.g., participation in counseling and using pharmacotherapies) and for being abstinent (e.g., 2 weeks, 2 months, 6 months, and 12 months follow-up). Patients received financial incentives at each of these visits. Written, informed consent was obtained from participants during enrollment. The protocol was approved by the institutional review board (protocol number 01494) and a description of the study protocol is available [7].

### Interventions and outcomes

Every patient enrolled received enhanced usual care. This included hospital-directed tobacco-use screening, counseling, education, and pharmacotherapy; all prescribed at the discretion of the patient’s health care team. Patients were interviewed at baseline, 2 weeks, 2 months, 6 months, and 12 months follow up. Patients’ sociodemographic data, smoking history, health, admitting service, and financial stress were taken at baseline. Follow up surveys assessed abstinence and adherence to counseling and smoking cessation pharmacotherapies. Smoking cessation for these time periods was bio confirmed with expired carbon monoxide (CO) or salivary cotinine at 2 months and salivary cotinine only at 6 months.

### Eligibility criteria and enrollment

Inclusion criteria were that patients had to be at least 18 years old, have smoked tobacco during the 30 days prior to hospitalization, have an active US phone number, reside in the New York City area, and be contemplating smoking cessation. Exclusion criteria were that if patients did not meet any of the inclusion criteria or if patients were anticipating discharge to an institution, such as a nursing home, with smoking restrictions. We maintained a log of all smoking patients based on the EMR screening already in place. This log was randomly regenerated every day to ensure equal distribution of enrollment targets across patient demographics and admitting services. The log kept data of enrolled vs non-enrolled patients along with their reason for not being enrolled (patient refusal, patient ineligible, patient unable to be approached) and basic demographic data but with no identifying information. If patients were unable to be enrolled because they were not approachable (e.g., poor mental status), they were re-approached later on in their stay. Overall, 929 patients were screened, 268 met eligibility criteria, and 182 were enrolled and randomized.

### Measures

The primary outcome for this secondary analysis was the official enrollment in the FIESTA trial among the eligible patients, coded as a binary variable (0 = not enrolled, 1 = enrolled). The exposure variable was the admitting services, which are Medicine, Psychiatry, and other. In this analysis, Medicine and other services were collapsed to ensure analytic stability due to a small proportion of participants admitted to other services. The other predictors included sex, age at baseline, race/ethnicity, education, and number of cigarettes per day.

### Statistical analyses

Descriptive statistics for the enrolled and non-enrolled groups were calculated to describe the enrollment rates among the eligible patients, with enrollment rate defined the number of enrolled patients divided by the total number of eligible patients. Baseline age was compared between groups using a two-sample t-test. Categorical variables were compared using χ^2^ tests or Fisher exact tests when expected cell counts < 5. Inspection of the dataset was done before primary analysis. To ensure the interpretability of the analysis, the types of missingness were identified based on study design – distinguishing missing at random or those due to branching logic. Multicollinearity was assessed prior to model fitting using variance inflation factors, and multicollinearity was not identified.

Minor missingness was observed for race/ethnicity (3.7%), cigarettes smoked per day (5.2%), and education (14.9%). Examination of missing-data patterns indicated a non-monotone pattern; 220 of 268 patients (82.1%) had complete data on all analytic variables. Among incomplete cases, education was most commonly missing, either alone (n = 24) or jointly with cigarettes smoked per day (n = 14); race/ethnicity was missing alone for 8 patients and jointly with education for 2 patients. Given the non-monotone pattern of missingness across multiple covariates and the inclusion of both continuous and categorical variables, missing data were imputed using multiple imputations by chained equations (MICE). Twenty imputed datasets were generated, each based on 50 iterations. For each imputed dataset, variable selection was carried out using Least Absolute Shrinkage and Selection Operator (LASSO) approach to build a predictive model for the primary analysis [8,9]. The candidate predictors considered in the LASSO procedure were admitting service, age, sex, race/ethnicity, education, and number of cigarettes smoked per day. LASSO with cross-validation was applied separately to each of the 20 imputed datasets, using the value of lambda that minimized the cross-validation error, to assess the stability of predictor selection across imputations. Because all six candidate predictors were prespecified as clinically relevant demographic or smoking-related factors and at least one coefficient for each predictor was retained by LASSO in nearly all or all imputed datasets, all six predictors were retained in the final multivariable logistic regression model.

Regression models were fitted separately in each imputed dataset, and estimates, 95% confidence intervals (CIs), and p-values were pooled using Rubin’s Rules. Fractions of missing information were also computed for each coefficient to quantify the loss of stability attributable to missing data. Separately, univariable logistic regression models were fitted for each candidate predictor within each of the 20 imputed datasets to estimate crude associations with enrollment. Crude odds ratios (cORs), 95% CIs, and p-values were pooled using Rubin’s Rules and are presented alongside the adjusted estimates in Table 2. The discrimination powers of the model was assessed using the area under the receiver operating characteristic curve (AUC) within each imputed dataset, and the mean AUC across the 20 imputed datasets was reported. All tests were two-sided, and p < 0.05 was considered statistically significant. P-values were not adjusted for multiple comparisons due to the exploratory nature of the study. Analyses were performed with R studio, version 2023.06.2. *MICE* R packages were used to inspect and impute datasets.

## Results

Participant screening, eligibility assessment, and enrollment are summarized in S1 Fig. Baseline characteristics of the hospitalized patients from the VA New York Harbor Healthcare System are presented in Table 1. There were 182 patients enrolled in the study and the majority were male (93.3%), non-Hispanic Black, (44.4%) followed by non-Hispanic White (30.6%). Over a third (35.1%) of the patients had a high school education or less.

**Table 1:** Characteristics and Enrollment Rate of Hospitalized Patients from the VA New York Harbor Healthcare System’s Manhattan campus from July 2015 to March 2018 (N = 268).

Variable	Total sample n (%)	Enrollment Rate (%)	Not enrolled (n = 86) (%)	Enrolled (n = 182) (%)	P-value
**Department**					<0.001
Internal Medicine/Other	162 (60.4)	59.9	65 (75.6)	97 (53.3)	
Psychiatry	106 (39.6)	80.2	21 (24.4)	85 (46.7)	
**Mean Age at baseline (SD)**	59.3 (11.5)	–	62.1 (10.8)	58.0 (11.7)	0.004
**Sex**					0.368
Male	250 (93.3)	68.8	78 (90.7)	172 (94.5)	
Female	18 (6.7)	55.6	8 (9.3)	10 (5.5)	
**Race/ethnicity**					0.049
White, non-Hispanic	76 (28.4)	56.6	33 (38.4)	43 (23.6)	
Black, non-Hispanic	114 (42.5)	72.8	31 (36.0)	83 (45.6)	
Hispanic	65 (24.3)	75.4	16 (18.6)	49 (26.9)	
Other	3 (1.1)	66.7	1 (1.2)	2 (1.1)	
Missing	10 (3.7)	50.0	5 (5.8)	5 (2.7)	
**Education**					0.004
High school or lower	94 (35.1)	87.2	12 (14.0)	82 (45.1)	
Associate/some college	108 (40.3)	77.8	24 (27.9)	84 (46.2)	
College graduate	26 (9.7)	57.7	11 (12.8)	15 (8.2)	
Missing	40 (14.9)	2.5	39 (45.3)	1 (0.5)	
**Cigarettes per day**					0.156
≤ 5	82 (30.6)	63.4	30 (34.9)	52 (28.6)	
>5 and ≤ 10	82 (30.6)	79.3	17 (19.8)	65 (35.7)	
> 10 and ≤ 20	77 (28.7)	71.4	22 (25.6)	55 (30.2)	
> 20	13 (4.9)	76.9	3 (3.5)	10 (5.5)	
Missing	14 (5.2)	0.0	14 (16.3)	0 (0.0)	

Enrollment status was coded as a binary variable (0 = did not participate, 1 = participated). Values are *n* (%) unless otherwise stated. Baseline age was compared between groups using a two-sample *t*-test. Categorical variables were compared using χ² tests or Fisher exact tests when expected cell counts < 5. “Missing” categories reflect observed missing data prior to imputation.

The enrollment rate varied by admitting service (Internal Medicine/other: 59.9%; Psychiatry: 80.2%). Patients with high school or lower education had the highest enrollment rate (87.2%) whereas that of college graduates was the lowest (57.7%). Those who smoked more than 5 and less than 10 cigarettes per day had the highest enrollment rate (79.3%).

Adjusted multivariable logistic regression results are presented in Table 2. Patients on the psychiatric service had a higher enrollment compared to those on the internal medicine service (aOR: 2.51 95% CI:1.23, 5.12). Older age was associated with lower odds of enrollment (aOR = 0.97, 95% CI: 0.94, 0.99). Those with a college degree had lower odds of enrollment compared to those with high school or lower (aOR: 0.17, 95% CI: 0.06, 0.49). Hispanic patients had higher odds of enrollment compared to non-Hispanic White patients (aOR: 2.52, 95% CI:1.10, 5.75). There were no significant discrepancies observed by sex or number of cigarettes smoked per day. Fractions of missing information (FMI) for model coefficients ranged from 0.02 to 0.29, suggesting that missingness contributed modestly to the uncertainty of the estimates. The model demonstrated moderate performance, with a mean AUC of 0.746 (SD = 0.013) across the 20 imputed datasets.

**Table 2:** Adjusted and crude logistic regression model examining associations between enrollment and variables among the hospitalized patients from the veterans affairs New York harbor healthcare system’s Manhattan campus.

Variable	aOR (95% CI)	P-value	FMI	cOR (95% CI)	Crude P-value
**Department**					
Internal Medicine/Other	Ref			Ref	
Psychiatry	2.51 (1.23, 5.12)	0.012	0.061	2.71 (1.53, 4.81)	0.001
**Age**	0.97 (0.94, 0.99)	0.025	0.253	0.97 (0.94, 0.99)	0.008
**Sex**					
Male	Ref			Ref	
Female	0.40 (0.12, 1.27)	0.120	0.019	0.57 (0.21, 1.50)	0.125
**Race/ethnicity**					
White, non-Hispanic	Ref			Ref	
Black, non-Hispanic	1.87 (0.96, 3.68)	0.067	0.064	2.07 (1.13, 3.80)	0.019
Hispanic	2.52 (1.11, 5.75)	0.028	0.078	2.39 (1.16, 4.93)	0.018
Other	2.29 (0.17, 31.43)	0.534	0.042	1.55 (0.13, 18.15)	0.728
**Education**					
High school or lower	Ref			Ref	
Associate/some college	0.44 (0.20, 1.12)	0.087	0.278	0.60 (0.30, 1.21)	0.153
College graduate	0.17 (0.06, 0.49)	0.001	0.285	0.22 (0.08, 0.57)	0.002
**Cigarettes per day**					
≤ 5	Ref			Ref	
>5 and ≤ 10	2.10 (0.99, 4.46)	0.053	0.068	2.29 (1.16, 4.51)	0.017
> 10 and ≤ 20	1.14 (0.53, 2.51)	0.753	0.141	1.43 (0.74, 2.77)	0.291
> 20	1.76 (0.40, 7.76)	0.451	0.102	2.02 (0.51, 8.01)	0.314

Abbreviations: aOR, Adjusted odds ratio, cOR, Crude odds ratio; CI: Confidence interval, FMI: Fraction of missing information

*Note.* FMI values correspond to estimates from the adjusted multivariable model. P-values for crude analyses are unadjusted and interpreted as exploratory given the secondary nature of this analysis.

## Discussion

We found that enrollment rates for a randomized controlled financial incentives study did vary significantly by admitting service. Those admitted to the Psychiatry unit were enrolled at higher rates than those admitted to the Internal Medicine unit. Education levels did show some significant variations in the enrollment rate. There was a higher enrollment rate among those who received a high school degree or lower compared to those who had a higher education level.

Patients enrolled from the Psychiatry department experienced significantly higher levels of enrollment (80.2%) than the Internal Medicine or other departments (59.9%). Further, there is a greater prevalence of smoking in psychiatric departments since, on average, patients with a psychiatric diagnosis are more likely to smoke [10]. Variations in diagnoses potentially impacts the degree of motivation these patients have to quit smoking. Higher enrollment rates, as demonstrated in this study, combined with a higher prevalence make psychiatric patients a group that could possibly benefit from additional focus of enrollment efforts.

Education was an unexpectedly significant factor in enrollment. We found that patients with a lower education had higher enrollment rates compared to those patients that had an associate or bachelor’s degree. Additionally, we found that Hispanic patients were enrolled at a significantly higher rate than White patients.

Our findings contribute to continually evolving efforts to include minorities in clinical research in order to develop clinical interventions that are generalizable to the public. Black patients represent 5% of research participants in medical journal publications, despite making up over 12% of the US population [11]. Minorities are often underrepresented in research because they are excluded from studies at a higher rate than whites due to not meeting inclusion criteria. With smoking cessation studies, these exclusion reasons typically include a concurrent diagnosis or attendance issues preventing minority patients from enrolling in studies [12]. While there has been recent investment into increasing representation of minorities in medical studies, reexamining these efforts reveals that there has been an overrepresentation of minorities in phase I trials and still a lack of representation in phase III trials. Diverse enrollment into later clinical studies such as phase III trials is essential to making generalizable conclusions on clinical interventions [13]. FIESTA was successful in its enrollment of a diverse population. Smoking cessation studies should include a diverse array of patient populations so that interventions can be generalizable to the smoking population. This paper supports the inclusion of patients from psychiatric admissions into such studies, but is limited in its mostly male patient population and that enrollment was from a single center. Findings may not be generalizable to non-VA, community based, female, or more diverse populations.

Further, there typically exists a disparity in the education level of patients enrolled to medical research. It has been found previously that patients with higher education levels and higher health literacy had higher levels of research interest and participation [14]. Because smoking cessation therapy intervention may be more easily understandable than other research interventions, health literacy limitations may not apply in the same way to smoking cessation trials; thus, smoking cessation trials signify a realm of scientific research in which lower educated patients can be better represented.

Our findings potentially demonstrate that Black patients were equally as likely to enroll as White patients. Additionally, patients with lower education are a typically underrepresented demographic to both enroll and educate around health and research. To the best of our knowledge, this is the first analysis of the effectiveness of enrollment amidst various admitting services for an urban hospital-based smoking cessation study.

Urban hospitals provide an opportune setting to address disparities in smoking cessation. Previous studies have utilized the urban hospital to enroll patients for studies on various smoking cessation technologies, medications, and counseling [15,16]. Understanding the patient demographics that yield higher enrollment rates is useful in planning efficient patient enrollment protocols. Our study indicates that the psychiatric service may yield more participants when designing enrollment protocols and choices based on education demographics could potentially streamline patient enrollment. Randomized trials are more likely to be representative of the overall smoking population if diverse populations are enrolled.

In analyzing the enrollment outcomes for the FIESTA study, we had limited access to the demographic information of those who were approached and declined to participate and those who were enrolled. Compared to the 182 participants enrolled, of the 268 patients screened for FIESTA, we only had access to the patients’ admitting service, age, sex, race, education, and smoking history. Other variables that would be interesting to compare enrollment rates for in future smoking cessation studies include health comorbidities and confidence to quit smoking. Of note, there was a higher representation of those with hypertension as compared to other comorbidities within the FIESTA study. If we had access to the degree of hypertension of the patients who declined to participate, determining what comorbidities seemingly motivate patients to participate in smoking cessation therapies and studies would be a useful metric for future public health efforts around smoking cessation. Additionally, As this was an exploratory study, no correction for multiple comparisons was performed, which may increase the risk of Type I error; therefore, findings should be interpreted as hypothesis-generating.

Ensuring that populations are diverse in smoking cessation studies allows research to be more generalizable to the smoking population. Research and clinical interventions directed towards those with mental health diagnoses would yield improved outcomes given the high interest and smoking prevalence among such patients.

## Supporting information

S1 Fig Supporting information. (DOCX)

S2 File Minimal data set. (XLSX)

S3 File Data dictionary. (XLSX)

## References

[1] Office of Disease Prevention and Health Promotion. Reduce current cigarette smoking in adults — TU-02. Washington (DC): US Department of Health and Human Services; 2023. https://odphp.health.gov/healthypeople/objectives-and-data/browse-objectives/tobacco-use/reduce-current-cigarette-smoking-adults-tu-02/infographic

[2] Houston TK, Scarinci IC, Person SD, Greene PG. Patient smoking cessation advice by health care providers: The role of ethnicity, socioeconomic status, and health. Am J Public Health. 2005;95(6):1056–61. doi: 10.2105/AJPH.2004.039909 15914833 PMC1449308

[3] Chhatre S, Jefferson A, Cook R, Meeker CR, Kim JH, Hartz KM, et al. Patient-centered recruitment and retention for a randomized controlled study. Trials. 2018;19(1):205. doi: 10.1186/s13063-018-2578-7 29587805 PMC5870194

[4] Bower P, Brueton V, Gamble C, Treweek S, Smith CT, Young B, et al. Interventions to improve recruitment and retention in clinical trials: A survey and workshop to assess current practice and future priorities. Trials. 2014;15:399. doi: 10.1186/1745-6215-15-399 25322807 PMC4210542

[5] Konyalıhatipoğlu EB, Karadoğan D, Telatar TG, Şahin Ü. Mid-term outcomes of a smoking cessation program in hospitalized patients in Türkiye. Tob Induc Dis. 2024;22:10.18332/tid/191239. doi: 10.18332/tid/191239 39081591 PMC11288063

[6] Ladapo JA, Tseng C-H, Sherman SE. Financial incentives for smoking cessation in hospitalized patients: A randomized clinical trial. Am J Med. 2020;133(6):741–9. doi: 10.1016/j.amjmed.2019.12.025 31982494 PMC7293955

[7] French KM, Gonzalez SZ, Sherman SE, Link AR, Malik SZ, Tseng C-H, et al. Financial IncEntives for Smoking TreAtment: Protocol of the FIESTA trial and FIESTA Oral Microbiome Substudy. Trials. 2018;19(1):646. doi: 10.1186/s13063-018-3003-y 30463608 PMC6249787

[8] Friedman J, Hastie T, Tibshirani R. Regularization paths for generalized linear models via coordinate descent. J Stat Softw. 2010;33(1):1–22. doi: 10.18637/jss.v033.i01 20808728 PMC2929880

[9] Tibshirani R. Regression shrinkage and selection via the lasso. J Royal Stat Soc Series B: Stat Methodol. 1996;58(1):267–88. doi: 10.1111/j.2517-6161.1996.tb02080.x

[10] Kalman D, Morissette SB, George TP. Co-morbidity of smoking in patients with psychiatric and substance use disorders. Am J Addict. 2005;14(2):106–23. doi: 10.1080/10550490590924728 16019961 PMC1199553

[11] Alegria M, Sud S, Steinberg BE, Gai N, Siddiqui A. Reporting of participant race, sex, and socioeconomic status in randomized clinical trials in general medical journals, 2015 vs 2019. JAMA Netw Open. 2021;4(5):e2111516. doi: 10.1001/jamanetworkopen.2021.11516 34037736 PMC8155820

[12] Webb Hooper M, Asfar T, Unrod M, Dorsey A, Correa JB, Brandon KO, et al. Reasons for exclusion from a smoking cessation trial: An analysis by race/ethnicity. Ethn Dis. 2019;29(1):23–30. doi: 10.18865/ed.29.1.23 30713413 PMC6343546

[13] Fisher JA, Kalbaugh CA. Challenging assumptions about minority participation in US clinical research. Am J Public Health. 2011;101(12):2217–22. doi: 10.2105/AJPH.2011.300279 22021285 PMC3222419

[14] Kripalani S, Goggins K, Couey C, Yeh VM, Donato KM, Schnelle JF, et al. Disparities in research participation by level of health literacy. Mayo Clin Proc. 2021;96(2):314–21. doi: 10.1016/j.mayocp.2020.06.058 33549253 PMC7874435

[15] Rigotti NA, Munafo MR, Stead LF. Interventions for smoking cessation in hospitalised patients. Cochrane Database Syst Rev. 2007;(3):CD001837. doi: 10.1002/14651858.CD001837.pub2 17636688

[16] Sherman SE, Link AR, Rogers ES, Krebs P, Ladapo JA, Shelley DR, et al. Smoking-cessation interventions for urban hospital patients: A randomized comparative effectiveness trial. Am J Prev Med. 2016;51(4):566–77. doi: 10.1016/j.amepre.2016.06.023 27647057 PMC5089173

