# Supplementary material for: An analysis of enrollment rates by admitting service for a secondary analysis of enrollment data from a randomized smoking-cessation trial (FIESTA)
Source: PLoS One. 2026 Nov 4;21(11):e0345267. doi: 10.1371/journal.pone.0345267 (PMC13734444; doi:10.1371/journal.pone.0345267)
Supplement: Supplementary file 1 [file pone.0345267.s001.docx]

Supplementary Figure 1. Flow of Participant Screening, Eligibility, and Randomization Across Medicine, Psychiatry, and Other Departments

*Supplementary Figure 1.* Flow diagram of participant screening, eligibility, and randomization across Medicine, Psychiatry, and other departments. Numbers represent participants assessed, excluded, eligible, declined participation, and randomized.

|  | **Dataset 1** | | **Dataset 2** | | **Dataset 3** | | **Dataset 4** | | **Dataset 5** | |
| --- | --- | --- | --- | --- | --- | --- | --- | --- | --- | --- |
|  | **aOR**  **(95% CI)** | **p-value** | **aOR**  **(95%CI)** | **p-value** | **aOR**  **(95% CI)** | **p-value** | **aOR**  **(95% CI)** | **p-value** | **aOR**  **(95% CI)** | **p-value** |
| **Department** |  |  |  |  |  |  |  |  |  |  |
| Medicine/Other | Ref |  | Ref |  | Ref |  | Ref |  | Ref |  |
| Psychiatry | 2.88 (1.43, 6.01) | 0.004 | 2.68 (1.35, 5.49) | 0.006 | 2.28 (1.18, 4.65) | 0.016 | 2.38 (1.21, 4.82) | 0.013 | 2.42 (1.23, 4.94) | 0.012 |
| **Age** | 0.97 (0.94, 0.99) | 0.021 | 0.97 (0.94, 1.01) | 0.022 | 0.97 (0.94, 1.01) | 0.024 | 0.97 (0.94, 0.99) | 0.021 | 0.97 (0.94, 1.00) | 0.028 |
| **Sex** |  |  |  |  |  |  |  |  |  |  |
| Male | Ref |  | Ref |  | Ref |  | Ref |  | Ref |  |
| Female | 0.37 (0.11, 1.18) | 0.090 | 0.42 (0.13, 1.37) | 0.150 | 0.40 (0.13, 1.26) | 0.110 | 0.41 (0.13, 1.30) | 0.130 | 0.41 (0.94, 1.34) | 0.140 |
| **Race/ethnicity** |  |  |  |  |  |  |  |  |  |  |
| White, non-Hispanic | Ref |  | Ref |  | Ref |  | Ref |  | Ref |  |
| Black, non-Hispanic | 2.08 (1.02, 4.26) | 0.044 | 2.20 (1.11, 4.41) | 0.024 | 2.06 (1.06, 4.04) | 0.034 | 2.11 (1.07, 4.18) | 0.031 | 2.46 (0.96, 4.08) | 0.055 |
| Hispanic | 2.35 (1.06, 5.34) | 0.037 | 2.53 (1.15, 5.71) | 0.022 | 2.31 1.08, 5.09) | 0.034 | 2.56 (1.17, 5.81) | 0.021 | 2.78 (1.28, 6.24) | 0.011 |
| Other | 2.59 (0.21, 64.7) | 0.500 | 2.52 (0.21, 60.9) | 0.500 | 2.13 (0.17, 54.4) | 0.600 | 2.38 (0.19, 59.4) | 0.500 | 2.47 (0.19, 62.7) | 0.500 |
| **Education** |  |  |  |  |  |  |  |  |  |  |
| High school or lower | Ref |  | Ref |  | Ref |  | Ref |  | Ref |  |
| Associate/some college credits | 0.30 (0.14, 0.61) | 0.001 | 0.36 (0.18, 0.70) | 0.003 | 0.57 (0.30, 1.08) | 0.089 | 0.40 (0.20, 0.77) | 0.008 | 0.51 (0.26, 0.99) | 0.050 |
| College graduate | 0.08 (0.03, 0.20) | < 0.001 | 0.11 (0.04, 0.28) | < 0.001 | 0.31 (0.12, 0.79) | 0.014 | 0.18 (0.07, 0.44) | <0.001 | 0.18 (0.07, 0.45) | < 0.001 |
| **Cigarettes per day** |  |  |  |  |  |  |  |  |  |  |
| ≤ 5 | Ref |  | Ref |  | Ref |  | Ref |  | Ref |  |
| >5 and ≤ 10 | 2.06 (1.00, 4.36) | 0.053 | 1.82 (0.88, 3.82) | 0.110 | 2.15 (1.08, 4.39) | 0.032 | 2.19 (1.07, 4.58) | 0.034 | 2.65 (1.29, 5.61) | 0.009 |
| > 10 and ≤ 20 | 1.18 (0.55, 2.55) | 0.700 | 0.98 (0.47, 2.05) | 0.900 | 1.26 (0.62, 2.61) | 0.500 | 1.10 (0.52, 2.31) | 0.800 | 1.38 (0.66, 2.92) | 0.400 |
| > 20 | 1.58 (0.42, 6.97) | 0.500 | 1.85 (0.47, 9.38) | 0.400 | 2.32 (0.61, 11.6) | 0.200 | 2.01 (0.53, 9.94) | 0.300 | 1.73 (0.49, 7.21) | 0.400 |

Supplementary Table 1. Adjusted Odds Ratios (aORs) and 95% Confidence Intervals From Logistic Regression Models Pooled Across 20 Imputed Datasets

|  | **Dataset 6** | | **Dataset 7** | | **Dataset 8** | | **Dataset 9** | | **Dataset 10** | |
| --- | --- | --- | --- | --- | --- | --- | --- | --- | --- | --- |
|  | **aOR**  **(95% CI)** | **p-value** | **aOR**  **(95%CI)** | **p-value** | **aOR**  **(95% CI)** | **p-value** | **aOR**  **(95% CI)** | **p-value** | **aOR**  **(95% CI)** | **p-value** |
| **Department** |  |  |  |  |  |  |  |  |  |  |
| Medicine/ Other | Ref |  | Ref |  | Ref |  | Ref |  | Ref |  |
| Psychiatry | 2.91 (1.45, 6.09) | 0.003 | 2.64 (1.32, 5.45) | 0.007 | 2.56 (1.31, 5.18) | 0.007 | 2.84 (1.43, 5.88) | 0.004 | 2.35 (1.20, 4.73) | 0.015 |
| **Age** | 0.97 (0.94, 1.00) | 0.041 | 0.97 (0.94, 1.00) | 0.036 | 0.97 (0.94, 0.99) | 0.021 | 0.97 (0.94, 1.00) | 0.038 | 0.97 (0.94, 1.00) | 0.027 |
| **Gender** |  |  |  |  |  |  |  |  |  |  |
| Male | Ref |  | Ref |  | Ref |  | Ref |  | Ref- |  |
| Female | 0.37 (0.11, 1.19) | 0.092 | 0.40 (0.12, 1.30) | 0.120 | 0.41 (0.13, 1.31) | 0.130 | 0.39 (0.12, 1.26) | 0.110 | 0.38 (0.12, 1.20) | 0.0.95 |
| **Race/ethnicity** |  |  |  |  |  |  |  |  |  |  |
| White, non-Hispanic | Ref |  | Ref |  | Ref |  | Ref |  | Ref |  |
| Black, non-Hispanic | 2.05 (0.99, 3.89) | 0.061 | 1.89 (0.94, 3.80) | 0.074 | 2.10 (1.06, 4.22) | 0.035 | 1.84 (0.92, 3.70) | 0.085 | 2.29 (1.16, 4.55) | 0.015 |
| Hispanic | 2.48 (1.14, 5.59) | 0.025 | 2.40 (1.08, 5.50) | 0.034 | 2.06 (0.95, 4.54) | 0.069 | 2.13 (0.99, 5.73) | 0.057 | 2.80 (1.28, 6.35) | 0.011 |
| Other | 2.55 (0.21, 61.6) | 0.500 | 2.21 (0.18, 54.1) | 0.500 | 2.60 (0.27, 60.0) | 0.500 | 2.23 (0.18, 54.6) | 0.500 | 2.58 (0.20, 64.9) | 0.500 |
| **Education** |  |  |  |  |  |  |  |  |  |  |
| High school or lower | Ref |  | Ref |  | Ref |  | Ref |  | Ref |  |
| Associate or some college credits | 0.48 (0.23, 0.92) | 0.029 | 0.50 (0.25, 0.99) | 0.049 | 0.47 (0.24, 0.92) | 0.029 | 0.49 (0.25, 0.95) | 0.0.38 | 0.48 (0.24, 0.92) | 0.029 |
| College graduate | 0.11 (0.05, 0.27) | < 0.001 | 0.13 (0.05, 0.30) | <0.001 | 0.15 (0.06, 0.36) | <0.001 | 0.15 (0.06, 0.36) | <0.001 | 0.19 (0.07, 0.45) | < 0.001 |
| **Cigarettes per Day** |  |  |  |  |  |  |  |  |  |  |
| ≤ 5 | Ref |  | Ref |  | Ref |  | Ref |  | Ref |  |
| >5 and ≤ 10 | 1.88 (0.90, 4.00) | 0.100 | 2.09 (1.01, 4.41) | 0.050 | 2.00 (0.97, 4.21) | 0.063 | 2.03 (0.98, 4.28) | 0.059 | 2.05 (1.01, 4.20) | 0.049 |
| > 10 and ≤ 20 | 0.83 (0.39, 1.73) | 0.600 | 1.16 (0.55, 2.48) | 0.700 | 0.93 (0.45, 1.96) | 0.900 | 0.92 (0.44, 1.93) | 0.800 | 1.32 (0.63, 2.79) | 0.500 |
| > 20 | 1.83 (0.46, 9.41) | 0.400 | 1.24 (0.34, 5.26) | 0.800 | 1.73 (0.44, 8.85) | 0.500 | 1.44 (0.37, 7.25) | 0.600 | 2.41 (0.61, 12.3) | 0.200 |

|  | **Dataset 11** | | **Dataset 12** | | **Dataset 13** | | **Dataset 14** | | **Dataset 15** | |
| --- | --- | --- | --- | --- | --- | --- | --- | --- | --- | --- |
|  | **aOR**  **(95% CI)** | **p-value** | **aOR**  **(95%CI)** | **p-value** | **aOR**  **(95% CI)** | **p-value** | **aOR**  **(95% CI)** | **p-value** | **aOR**  **(95% CI)** | **p-value** |
| **Department** |  |  |  |  |  |  |  |  |  |  |
| Medicine/ Other | Ref |  | Ref |  | Ref |  | Ref |  | Ref |  |
| Psychiatry | 2.43 (1.24, 4.92) | 0.011 | 2.24 (1.15, 4.47) | 0.020 | 2.52 (1.28, 5.11) | 0.008 | 2.73 (1.38, 5.57) | 0.005 | 2.43 (1.25, 4.88) | 0.011 |
| **Age** | 0.97 (0.94, 1.00) | 0.0.36 | 0.96 (0.94, 0.99) | 0.016 | 0.97 (0.94, 0.99) | 0.021 | 0.97 (0.94, 0.99) | 0.021 | 0.96 (0.93, 0.99) | 0.013 |
| **Sex** |  |  |  |  |  |  |  |  |  |  |
| Male | Ref |  | Ref |  | Ref |  | Ref |  | Ref |  |
| Female | 0.35 (0.11, 1.11) | 0.070 | 0.39 (0.12, 1.25) | 0.110 | 0.40 (0.13, 1.28) | 0.120 | 0.42 (0.13, 135) | 0.140 | 0.43 (0.14, 1.38) | 0.200 |
| **Race/ethnicity** |  |  |  |  |  |  |  |  |  |  |
| White, non-Hispanic | Ref |  | Ref |  | Ref |  | Ref |  | Ref |  |
| Black, non-Hispanic | 2.36 (1.20, 4.68) | 0.013 | 2.40 (1.23, 4.76) | 0.011 | 2.19 (1.12, 4.35) | 0.023 | 1.94 (0.99, 3.83) | 0.054 | 2.30 (1.16, 4.62) | 0.018 |
| Hispanic | 2.80 (1.31, 6.17) | 0.001 | 3.03 (1.41, 6.78) | 0.006 | 2.90 (1.32, 6.63) | 0.010 | 2.45 (1.13, 5.49) | 0.027 | 2.45 (1.12, 5.48) | 0.026 |
| Other | 2.47 (0.20, 60.6) | 0.500 | 2.52 (0.20, 62.5) | 0.500 | 2.46 (0.19, 62.5) | 0.500 | 1.03 (0.11, 9.83) | 0.990 | 3.24 (0.33, 75.5) | 0.400 |
| **Education** |  |  |  |  |  |  |  |  |  |  |
| High school or lower | Ref |  | Ref |  | Ref |  | Ref |  | Ref |  |
| Associate/some college credits | 0.70 (0.36, 1.34) | 0.300 | 0.48 (0.24, 0.91) | 0.026 | 0.43 (0.21, 0.82) | 0.013 | 0.40 (0.20, 0.77) | 0.007 | 0.33 (0.13, 0.64) | 0.001 |
| College graduate | 0.19 (0.08, 0.45) | < 0.001 | 0.19 (0.07, 0.46) | < 0.001 | 0.18 (0.07, 0.43) | < 0.001 | 0.13 (0.11, 0.37) | < 0.001 | 0.20 (0.09, 0.51) | < 0.001 |
| **Cigarettes per day** |  |  |  |  |  |  |  |  |  |  |
| ≤ 5 | Ref |  | Ref |  | Ref |  | Ref |  | Ref |  |
| >5 and ≤ 10 | 1.83 (0.89, 3.79) | 0.100 | 1.88 (0.93, 3.86) | 0.083 | 2.28 (1.11, 4.80) | 0.026 | 2.12 (1.02, 4.47) | 0.045 | 2.28 (1.11, 4.77) | 0.026 |
| > 10 and ≤ 20 | 1.16 (0.56, 2.41) | 0.700 | 1.35 (0.65, 2.86) | 0.400 | 1.25 (0.60, 2.61) | 0.500 | 1.02 (0.49, 2.10) | 0.933 | 1.11 (0.54, 2.33) | 0.800 |
| > 20 | 2.01 (0.51, 10.2) | 0.300 | 1.22 (0.36, 4.57) | 0.800 | 1.50 (0.42, 2.82) | 0.500 | 1.57 (0.44, 6.62) | 0.500 | 2.09 (0.54, 10.4) | 0.300 |

|  | **Dataset 16** | | **Dataset 17** | | **Dataset 18** | | **Dataset 19** | | **Dataset 20** | |
| --- | --- | --- | --- | --- | --- | --- | --- | --- | --- | --- |
|  | **aOR**  **(95% CI)** | **p-value** | **aOR**  **(95%CI)** | **p-value** | **aOR**  **(95% CI)** | **p-value** | **aOR**  **(95% CI)** | **p-value** | **aOR**  **(95% CI)** | **p-value** |
| **Department** |  |  |  |  |  |  |  |  |  |  |
| Medicine/ Other | Ref |  | Ref |  | Ref |  | Ref |  | Ref |  |
| Psychiatry | 2.31 (1.19, 4.62) | 0.015 | 2.33 (1.25, 4.64) | 0.014 | 2.61 (1.32, 5.37) | 0.007 | 2.47 (1.27, 5.02) | 0.010 | 2.30 (1.17, 4.63) | 0.017 |
| **Age** | 0.97 (0.94, 0.99) | 0.021 | 0.97 (0.94, 0.99) | 0.017 | 0.97 (0.94, 1.00) | 0.035 | 0.96 (0.94, 0.99) | 0.014 | 0.96 (0.94, 0.99) | 0.017 |
| **Sex** |  |  |  |  |  |  |  |  |  |  |
| Male | Ref |  | Ref |  | Ref |  | Ref |  | Ref |  |
| Female | 0.43 (0.14, 1.38) | 0.400 | 0.40 (0.13, 1.27) | 0.120 | 0.36 (0.11, 1.14) | 0.080 | 0.42 (0.13, 1.33) | 0.150 | 0.41 (0.13, 1.30) | 0.130 |
| **Race/ethnicity** |  |  |  |  |  |  |  |  |  |  |
| White, non-Hispanic | Ref |  | Ref |  | Ref |  | Ref |  | Ref |  |
| Black, non-Hispanic | 2.27 (1.15, 4.52) | 0.018 | 1.87 (0.95, 3.71) | 0.069 | 2.09 (1.06, 4.15) | 0.033 | 2.20 (1.11, 4.41) | 0.025 | 2.00 (0.99, 4.06) | 0.053 |
| Hispanic | 2.78 (1.28, 6.29) | 0.012 | 2.24 (1.05, 4.95) | 0.041 | 2.67 (1.22, 6.04) | 0.015 | 2.77 (1.27, 6.25) | 0.012 | 2.23 (1.02, 5.02) | 0.048 |
| Other | 2.44 (0.19, 61.9) | 0.500 | 2.12 (0.17, 52.9) | 0.600 | 2.46 (0.20, 61.2) | 0.500 | 2.45 (0.19, 61.9) | 0.500 | 1.44 (0.14, 15.0) | 0.700 |
| **Education** |  |  |  |  |  |  |  |  |  |  |
| High school or lower | Ref |  | Ref |  | Ref |  | Ref |  | Ref |  |
| Associate/some college credits | 0.42 (0.20, 0.80) | 0.010 | 0.44 (0.23, 0.85) | 0.015 | 0.56 (0.29, 1.07) | 0.083 | 0.33 (0.16, 0.66) | 0.002 | 0.43 (0.22, 0.84) | 0.014 |
| College graduate | 0.20 (0.08, 0.49) | < 0.001 | 0.20 (0.08, 0.50) | < 0.001 | 0.18 (0.07, 0.43) | < 0.001 | 0.15 (0.06, 0.38) | < 0.001 | 0.18 (0.07, 0.44) | < 0.001 |
| **Cigarettes per day** |  |  |  |  |  |  |  |  |  |  |
| ≤ 5 | Ref |  | Ref |  | Ref |  | Ref |  | Ref |  |
| >5 and ≤ 10 | 2.37 (1.16, 4.94) | 0.019 | 2.15 (1.05, 4.48) | 0.038 | 2.03 (1.00, 4.35) | 0.053 | 2.10 (1.02, 4.41) | 0.0.47 | 2.22 (1.09, 4.64) | 0.030 |
| > 10 and ≤ 20 | 1.28 (0.62, 2.67) | 0.500 | 1.03 (0.50, 2.13) | 0.900 | 1.09 (0.53, 2.26) | 0.800 | 1.22 (0.57, 2.61) | 0.600 | 1.33 (0.63, 2.81) | 0.400 |
| > 20 | 2.39 (0.60, 12.2) | 0.200 | 1.82 (0.48, 8.95) | 0.400 | 2.16 (0.55, 11.0) | 0.300 | 1.12 (0.34, 3.92) | 0.900 | 2.06 (0.52, 10.5) | 0.300 |

Abbreviations: OR, Adjusted odds ratio, CI: Confidence interval

Note. P-values are unadjusted and interpreted as exploratory given the secondary nature of this analysis.

Supplementary Table 2. Adjusted Odds Ratios (aORs) and 95% Confidence Intervals from Logistic Regression Models Pooled Across 20 Imputed Datasets Without Participants in Other Department.

| **Variable** | **aOR**  **(95% CI)** | **P-value** | **FMI** |
| --- | --- | --- | --- |
| **Department** |  |  |  |
| Medicine | Ref |  |  |
| Psychiatry | 1.96 (1.01, 4.05) | 0.041 | 0.045 |
| **Age** | 0.95 (0.92, 0.99) | 0.008 | 0.025 |
| **Sex** |  |  |  |
| Male | Ref |  |  |
| Female | 0.31 (0.09, 1.03) | 0.06 | 0.025 |
| **Race/ethnicity** |  |  |  |
| White, non-Hispanic | Ref |  |  |
| Black, non-Hispanic | 2.41 (0.97, 4.24) | 0.067 | 0.109 |
| Hispanic | 2.19 (0.94, 5.12) | 0.070 | 0.085 |
| Other | 1.99 (0.14, 29.29) | 0.613 | 0.011 |
| **Education** |  |  |  |
| High school or lower | Ref |  |  |
| Associate/some college | 0.55 (0.25, 1.22) | 0.142 | 0.207 |
| College graduate | 0.20 (0.07, 0.57) | 0.003 | 0.175 |
| **Cigarettes per day** |  |  |  |
| ≤ 5 | Ref |  |  |
| >5 and ≤ 10 | 2.02 (0.89, 4.60) | 0.093 | 0.127 |
| > 10 and ≤ 20 | 1.35 (0.69, 3.06) | 0.465 | 0.083 |
| > 20 | 1.79 (0.40, 8.09) | 0.449 | 0.091 |

Abbreviations: aOR, Adjusted odds ratio, CI: Confidence interval, FMI: Fraction of missing information

Note. P-values are unadjusted and interpreted as exploratory given the secondary nature of this analysis.
