# Supplementary material for: An analysis of enrollment rates by admitting service for a secondary analysis of enrollment data from a randomized smoking-cessation trial (FIESTA)
Source: PLoS One. 2026 Nov 4;21(11):e0345267. doi: 10.1371/journal.pone.0345267 (PMC13734444; doi:10.1371/journal.pone.0345267)
Supplement: Supplementary file 2 [file pone.0345267.s005.docx]

Reviewer #1: I read the paper with an interest. It seems like descriptive study. There is an important finding to point, the higher recruitment rates of patients refered from psychiatry service. On the other hand sone previous studies have pointed that physiciatrist are the least motivated group to give smoking cessation service or teach tobacco related lectures, due to the relaxing effect on, seen in the following article ( DOI: 10.4103/1357-6283.178602, Education for Health • Volume 28 • Issue 3 (December 2015) )‘The two lecturers who were unconvinced that tobacco merited special attention were from the psychiatry and pharmacology departments. While one of two faculty members interviewed from the psychiatry department was in favor of using tobacco as an exemplar for teaching students about the process of addiction, a second psychiatrist was of the opinion that smoking was a useful coping mechanism for patients suffering from conditions such as anxiety, depression, and schizophrenia. ‘

Response: Thank you for this thoughtful feedback. We are grateful for your interest in our work and have addressed your comments below.


-Line 51:…in 2021,…where?? (in general or worldwide or US)

Response: We have updated the data to be from the 2023 NHIS publication as suggested by the second reviewer, and have clarified that the data refers to US smoking rates.

-Line 65: Please add reference for examples of hospital-based programmes, such as the following recent paper: DOI: 10.18332/tid/191239

Response: Thank you for this suggestion, I have included this interesting study as an example of a hospital-based smoking cessation program

-In table 1: ‘Medicine’ as name of department is not clear, which department of medicine, (internal medicine or surgery departments, or pulmonology or cardiology?)

Response: I have clarified that Medicine refers to Internal Medicine, both in the tables and as it appears in the results and discussion sections.

Reviewer #2: This secondary analysis of recruitment data from the FIESTA RCT addresses a practical but under-studied question: does admitting service (Medicine vs. Psychiatry) influence enrolment in an inpatient smoking-cessation trial? The manuscript is clearly written, the dataset is unusual, and the findings could inform recruitment planning. Nevertheless, methodological transparency and completeness of reporting require attention before the paper reaches PLOS ONE standards.

MAJOR REVISIONS
Study design mis-labelling
– Title page & Abstract, l. 3-4: Replace “a randomized control smoking cessation study” with “a secondary analysis of recruitment data from a randomized smoking-cessation trial (FIESTA).”

Response: Thank you. I have updated the title as suggested to more clearly represent the paper

– Introduction, l. 46-48: Add explicit sentence: “The present work is a secondary analysis of the FIESTA RCT (NCT02506829).”

Response: Thank you. I have added the suggest line to the abstract

CONSORT flow diagram missing
– Insert Figure 1 (after l. 88) showing screened → eligible → approached → enrolled, with reasons for non-enrolment, stratified by Medicine / Psychiatry / Other.

Response: Thank you for this helpful suggestion. We added a CONSORT flow diagram (Supplementary Figure 1) summarizing the numbers screened, eligible, approached, and enrolled, with reasons for non-enrollment, stratified by Medicine, Psychiatry, and other services.

Sparse “Other” admitting service
– Methods, l. 117-119 & Table 2: Only 22 patients (8 %) yield an imprecise aOR 0.71 (95 % CI 0.25–2.04).
– Collapse “Other” into “Medicine” or exclude and supply sensitivity analysis (supplement or appendix).

Response: Thank you for pointing this out. We collapsed the “Other” admitting service (n = 22) with “Medicine” in all analyses to improve model stability and clarity. This change is reflected in the Methods, Table 1, and Table 2. Significance remained unchanged when excluding the patients from “Other” department in the sensitivity analysis (see Supplementary Table 2).

Multiple-imputation reporting incomplete
– Methods, l. 137-143: State: (a) m = 20 imputed datasets, (b) 50 burn-in iterations, (c) predictor list (age, sex, race, education, cigarettes/day, service, LOS), (d) fraction of missing information (FMI) for each coefficient.

Response: Thank you for this important feedback. We have added the requested items to the methods section as bellows:

“Twenty imputed datasets were generated, each based on 50 iterations. For each imputed dataset, variable selection was carried out using Least Absolute Shrinkage and Selection Operator (LASSO) approach to build a predictive model for the primary analysis.”

“The other predictors included sex, age at baseline, race/ethnicity, education, and number of cigarettes per day.”

“Fractions of missing information were also computed for each coefficient to quantify the loss of stability attributable to missing data.”

– Clarify why education “Missing” (n = 40, Table 1) is excluded from adjusted model (l. 148).

Response: Thank you for noting this. For education, race/ethnicity and smoking history “Missing” values were imputed before fitting the model and therefore were not included as visible category in the adjusted model.

Confounding control
– Age and length-of-stay (LOS) are available in FIESTA baseline files but not used. Add both covariates to the final model or explain their absence (Discussion, l. 190-192).

Response: Thank you for this important feedback. We revised the entire analysis by adding Age variable. However, LOS was only found available among the patients who were enrolled in the study. Since our study compared enrolled versus non-enrolled, LOS could not be included.

Multiplicity of tests
– Footnote to Table 2: Apply Holm–Bonferroni correction across 20 pairwise tests; report adjusted p-values or label comparison “exploratory.”

Response: Thank you for this thoughtful suggestion. Since our study is a secondary analysis of recruitment rates, we aimed to present the effect sizes rather than testing pre-specified hypotheses, we did not apply family-wise error procedures. We have labeled all comparisons as exploratory and interpreted cautiously in the footnote of Table 2 as “P-values are unadjusted and interpreted as exploratory given the secondary nature of this analysis”.

Data-availability statement
– l. 245-247: Replace current sentence with:
“De-identified participant data are available from the VA New York Harbor Healthcare System for researchers who obtain the necessary VA data-use agreement (contact[scott.sherman@nyulangone.org](mailto:scott.sherman@nyulangone.org)).”

Response: Thank you for this suggestion. The lines suggested in this comment are within the references so I have added the suggested statement to the Study Design section of methods instead.

MINOR REVISIONS
Abstract results, l. 33-35: Add 95 % CIs for Psychiatry vs Medicine (3.20, 1.63–6.26) and high-school vs college (0.19, 0.07–0.51).

Response: Thank you for this suggestion, confidence intervals have been added to the abstract results.

Table 1 footnote, l. 154: Use χ² for cells ≥5; Fisher exact only for cells <5; be consistent.

Response: This has been addressed in the captions for supplemental figures and the footnote has been taken out.

Language, l. 198: Change “make studies generalizable” to “may improve representativeness.”

Response: Thank you for this suggestion. We hope to keep the wording to generalization rather than representation. Our aim is to make sure smoking-cessation studies are applicable to the general public, not to sway representation in studies to certain demographics.

References, l. 250: Update CDC smoking prevalence to 2023 NHIS (11.5 % adults).
Response: I have updated the source to one from the Office of Disease Prevention and Health Promotion that utilizes the NHIS 11.5% statistic for a more updated report than the 2021 CDC one I have previously included.

Supporting Information: Upload (a) CONSORT flow diagram, (b) R script (LASSO + mice code), (c) sensitivity analysis without “Other” service.

Response: The requested supporting information has been added.
