# Supplementary material for: An analysis of enrollment rates by admitting service for a secondary analysis of enrollment data from a randomized smoking-cessation trial (FIESTA)
Source: PLoS One. 2026 Nov 4;21(11):e0345267. doi: 10.1371/journal.pone.0345267 (PMC13734444; doi:10.1371/journal.pone.0345267)
Supplement: Supplementary file 3 [file pone.0345267.s006.docx]

Ref: PONE-D-25-20584

Dear Dr. Jennifer Tucker,

Thank you for extending the opportunity to revise our manuscript. We appreciate your comments and have carefully considered your feedback and made revisions accordingly. We have provided a comprehensive response addressing all the reviews and incorporated both reviewers’ feedback into our revision.

This paper has not been submitted or published elsewhere. All authors approved the final manuscript. There are no conflicts of interest. We appreciate your interest in our work.

Sincerely,

Kayla Koffler, MSIII

Reviewer #2:

1. Please state explicitly in the Introduction that this manuscript is a secondary analysis. The Abstract now notes that this is a secondary analysis of the FIESTA RCT, which is helpful. However, this should also be stated clearly in the Introduction so that the study design is explicit at the outset of the main text. A brief sentence identifying this work as a secondary analysis of recruitment data from the FIESTA trial (NCT02506829) would address this.

Response: Thank you for this feedback, we have added this into the introduction (lines 81-82)

2. Please reconcile the race/ethnicity findings across the Abstract, Results, Discussion, and Table 2. At present, the Abstract states that race did not significantly impact recruitment. However, Table 2 reports a statistically significant association for Hispanic participants compared with non-Hispanic White participants, and the Results/Discussion also describe higher recruitment among Hispanic patients. These statements should be made consistent throughout the manuscript. If the intent is to emphasize the exploratory nature of the subgroup comparisons, that should be stated clearly, but the wording should still accurately reflect the reported estimates.

Response: Thank you for this important observation. We have revised the inconsistencies and made statements aligning with the table results throughout the manuscript.

3. Please align the stated objective with the analyses presented. The Introduction states that the primary objective was to determine whether recruitment and retention were higher on the Medical Service than on the Psychiatry Service. However, the manuscript as currently written appears to analyze recruitment/enrollment only. Please revise the objective language accordingly, unless retention analyses are also intended to be included.

Response: Thank you for this suggestion. We revised the primary objective language to focus on recruitment as retention analyses were not performed in the present manuscript.

4. Please clarify the Data Availability Statement and ensure consistency across submission materials. The manuscript text states that de-identified participant data are available through the VA system with the appropriate data-use agreement, whereas the submission materials indicate that all relevant data are within the manuscript and Supporting Information files. Please provide one clear and accurate Data Availability Statement that reflects exactly what is publicly available and what, if anything, remains under restricted access.

The submission materials are correct – all relevant data are within the manuscript and Supporting Information files.

5. Please moderate the language regarding generalizability. The manuscript makes an important contribution by showing successful recruitment of groups often underrepresented in clinical research. However, some statements about making studies more “generalizable to the smoking population” remain stronger than warranted for a single-site VA study with a predominantly male sample. I suggest slightly tempering this language and distinguishing more clearly between inclusiveness within this study context and broader external validity.

Response: I believe that the language here is emphasizing the importance of the goal of making smoking cessation trials more generalizable to the public, not stating that this paper will facilitate that generalizability. I added in a line more clearly stating the limits of this study in that mission (lines 235-237)

6. Please revise the Discussion for consistency regarding which variables were available among screened patients. The Discussion states that, among screened patients, the authors only had access to admitting service, sex, race, education, and smoking history. However, age was included in the adjusted model and reported in the Results. Please revise this description so it is consistent with the variables actually used in the analysis.

Response: We revised the description in the discussion to include age as a variable we had access to (line 261)

7. Abstract Methods: please include age among the predictors. The Abstract Methods currently list admitting service, sex, race/ethnicity, education, and cigarettes per day, but age was also included in the adjusted model and was statistically significant. Please add age to the Abstract Methods for completeness and consistency.

Response: We appreciate the important observation. We added age to the Abstract Methods to ensure completeness and consistency with the primary analyses.

8. Please cite the recruitment flow diagram explicitly in the main text. The addition of the recruitment/CONSORT-style flow diagram is appreciated. Please ensure that it is explicitly referenced in the main text so that readers can readily identify it.

Response: Thank you for the suggestion. We included a citation of “Participant screening, eligibility assessment, and enrollment are summarized in Supplementary Figure 1.” in the Results section within the main text of the manuscript. We also added “Overall, 929 patients were screened, 268 met eligibility criteria, and 182 were randomized” in the eligibility criteria section to briefly describe the recruitment flow.

9. Please ensure that the ethics information is consistent across the manuscript and submission system. The manuscript indicates that the protocol received institutional review board approval, whereas the submission metadata appears to list the ethics statement as “N/A.” Please verify and correct this so that the ethics information is consistent across all submission components.

The FIESTA trial received IRB approval, which would cover these analyses. So, the correct answer should be that it was approved (rather than “N/A”).


10. Please conduct a final editorial consistency check. Before final acceptance, I recommend a final review for consistency in terminology, formatting, and numerical reporting across the manuscript, tables, and supplementary materials.

Response: Thank you for this suggestion, we have conducted several final read throughs for consistency.

Reviewer #4:

Line 30: The term secondary analyses is to be used.

Response: Updated the terminology to read secondary analysis.

Line 87: Allocation concealment and blinding are to be described.

Response: Thank you for the helpful suggestion. We added “Participants and study staff were not blinded to treatment assignment” to the main text within the manuscript to clarify the allocation concealment and blinding for the study.

Line 89: When are the financial incentives provided, at each visit or at the end of the study? Are these funded through research grants?

Response: We have added a clarification to the timing and funding (lines 97-98)

Line 117: The exclusion criteria is to be added.

Response: Exclusion criteria has been added.

The percentage of missing data to be stated along with the missing data pattern.

Response: Thank you for the comments. We added “Missingness ranged from 3.7% to 14.9% across all covariates, with the highest missingness observed for education and smoking history variables” along the missing data pattern description in the analysis section.

Line 140-141: The sentence requires revision e.g. After LASSO-based variable selection, regression models were fitted, and the estimates were pooled along with 95% confidence intervals (CIs) and p-values using Rubin’s Rules.

Response: Thank you for the comment. The sentence was revised for clarity now stated “After LASSO-based variable selection, regression models were fitted, and the estimates were pooled along with 95% confidence intervals (CIs) and p-values using Rubin’s Rules” in the Statistical Analyses section.

Line 127: The word Analysis to be replaced with Statistical Analyses.

Response: Thank you for the suggestion. The heading was replaced with Statistical Analyses for clarity purpose.

The statistical tests employed in the results section are to be clearly stated in the statistical analyses section.

Response: Thank you for this important note. We included “Baseline age was compared between groups using a two-sample t-test. Categorical variables were compared using χ² tests or Fisher exact tests when expected cell counts < 5” to describe the descriptive analyses. For the inferential analyses, LASSO approach and multivariable logistic regression were addressed in the Statistical Analyses section as suggested in the previous comments.

Line 156: Multivariable results? The sentence requires revision.

Response: We appreciate this important observation. The sentence was revised to “Adjusted multivariable logistic regression results are presented in Table 2” for completeness.

Table 1: Where applicable, percentage figure is to be included.

Response: Thank you for this helpful comment. Column percentages were added to Table 1 to provide comparability that raw frequencies alone cannot offer.

Line 171: The coding variable for the recruitment (outcome variable) is to be specified.

Response: Thank you for the observation. A statement of “The outcome was coded as a binary outcome variable (0 = did not participate, 1 = participated)” was added to both Methods section and footnote in Table 2.

Tables 1 and 2: For smoking history/cigarettes per day respectively, the categories overlap (e.g., someone who smokes 4 cigarettes fits both <5 and <10). It should be mutually exclusive. Smoking can be categorized into mutually exclusive groups (0–5, 6–10, 11–20, >20 cigarettes/day), with 0–5 cigarettes/day as the reference category as highlighted in the supplementary tables. Symbol * is to be clearly denoted in the table footnote.

Response: Thank you for the comment. The categories under cigarettes per day variable were mutually exclusive throughout the analyses. The labels of categories were revised to ≤ 5, >5 and ≤ 10, > 10 and ≤ 20, and > 20 for less confusion. The Symbol * has been removed from both Table 1 and Table 2.

Table 2: Crude (unadjusted) analyses are to be presented alongside adjusted analyses. The model selection strategy, handling of confounders (selection criteria), and assessment of model fit are to be described. Column size for FMI could be reduced.

Response: We appreciate this comment. Crude odds ratio was added to Table 2 along with a p-value. The Statistical Analyses was further revised to “Missingness ranged from 3.7% to 14.9% across all covariates, with the highest missingness observed for education and smoking history variables. Missing data was imputed using multiple imputations by chained equations (MICE). Twenty imputed datasets were generated, each based on 50 iterations. For each imputed dataset, variable selection was carried out using Least Absolute Shrinkage and Selection Operator (LASSO) approach to build a predictive model for the primary analysis. Variables consistently retained across all imputed datasets were included in the final multivariable logistic regression model. Demographic and smoking-related variables were considered as potential confounders based on clinical relevance. After LASSO-based variable selection, regression models were fitted, and the estimates were pooled along with 95% confidence intervals (CIs) and p-values using Rubin’s Rules. Fractions of missing information were also computed for each coefficient to quantify the loss of stability attributable to missing data. No adjustments were made for multiple comparisons due to the exploratory nature of the study. All statistics tests were 2-sided with p-value < 0.05 testing significance. Analyses were performed with R studio, version 2023.06.2. *MICE* R packages were used to inspect and impute datasets” for more details to describe model selection, handling of confounders and assessment of model. The formatting of the FMI column in Table 2 was adjusted as suggested.

Line 177: To state P value (unadjusted for multiple comparisons).

Response: Thank you for the comment. “P-values are unadjusted and interpreted as exploratory given the secondary nature of this analysis” was added to the footnote in Table 2.

A statement ‘No adjustments were made for multiple comparisons due to the exploratory nature of the study’ to be included in the method section.

Response: Thank you for the important suggestion. This statement was added to the Statistical Analyses section in the Methods.

The fulfillment of the statistical assumptions, such as multicollinearity, etc., is to be stated.

Response: Thank you for the comment. We added “Multicollinearity was assessed prior to model fitting using variance inflation factors, and multicollinearity was not identified” to address the fulfilment of the statistical assumption before the final model estimation.

Supplementary Table 1: symbol <= is to be replaced with ≤.

Response: Thank you for this helpful comment. The symbol was corrected throughout the main manuscript as well as the supplementary materials.

Supplementary Table 1 and Supplementary Table 2: If the P values here are unadjusted for multiple comparisons, it is to be clearly stated in the table footnote.

Response: Thank you for this important observation. Footnotes in all supplementary tables were revised to clarify that p-values were unadjusted for multiple comparisons.

The primary objective ‘determine whether rates of FIESTA participant recruitment and retention were higher on the Medical Service than on the Psychiatry Service.’ is leaning towards hypothesis testing. While multiple comparisons were performed and since no adjustment was applied, this may increase the risk of Type I error and should be acknowledged or justified. If the study is an exploratory nature, it should be stated clearly and reflected in the manuscript e.g. introduction, method, discussion.

Response: Thank you for this observation, I have made note of this Type I error risk in the introduction (lines 82-84) and discussion (lines 268-270).

Some references did not conform to the journal’s formatting requirements.

Response: Thank you for pointing this out, we have updated the references to better fit with NLM/ICMJE style formatting.
