# Supplementary material for: An analysis of enrollment rates by admitting service for a secondary analysis of enrollment data from a randomized smoking-cessation trial (FIESTA)
Source: PLoS One. 2026 Nov 4;21(11):e0345267. doi: 10.1371/journal.pone.0345267 (PMC13734444; doi:10.1371/journal.pone.0345267)
Supplement: Supplementary file 4 [file pone.0345267.s007.docx]

Reviewer #2

**Major comments**

*1. Allocation concealment: The authors describe lack of blinding, but allocation concealment for the parent FIESTA trial remains unclear. Please briefly describe the randomization/allocation concealment procedure or cite the parent trial protocol/publication. If not relevant to this recruitment analysis, please state this explicitly.*

Response: The randomization procedure is not relevant to this study as we are solely commenting on the recruitment process, added in as (Lines 100-101 in the tracked changes version).

*2. Outcome definition: Please clearly define the binary outcome used in the logistic regression. Does “participation” mean consent, enrollment, randomization, or baseline visit attendance? Use the same term consistently throughout the manuscript.*

Response: We have clarified that primary outcome was enrollment in the FIESTA trial among eligible patients (0 = not enrolled and 1 = enrolled) in the Methods. We also revised the Results and tables to use “enrollment rate” for the proportion of eligible patients enrolled and “odds of enrollment” when reporting logistic regression results (Methods, Lines 137-139; Results, Lines 214-215; Table 1, Line 226).

We have replaced the terminology of recruitment with enrollment consistently throughout the paper.

*3. Missing data: The manuscript reports the percentage range of missing data, but not the missing-data pattern. Please describe whether missingness was monotone or non-monotone, whether variables were commonly missing together, and why MICE was considered appropriate.*

Response: We have expanded the Statistical Analyses section to describe the pattern of missing data and clarify the rationale for using MICE. We clarified that MICE was used given the non-monotone pattern of missingness across multiple covariates and its ability to accommodate the continuous and categorical variables included in the analysis (Methods, Lines 163-166).

*4. Model selection and confounding: The LASSO variable-selection procedure needs clearer description. Please specify candidate variables, the threshold for being “consistently retained” across imputed datasets, and how clinically important confounders were handled if not selected by LASSO.*

Response: We have revised the Statistical Analyses section to clarify the LASSO variable-selection procedure, including the candidate predictors and how selection across the imputed datasets was evaluated. We also clarified that all prespecified clinically relevant demographic and smoking-related covariates were retained in the final multivariable model; therefore, no clinically important candidate confounders were excluded based on LASSO selection (Methods, Lines 168-177).

*5. Model fit: VIF and fraction of missing information do not assess logistic regression model fit. Please report appropriate model performance/fit measures, such as C-statistic/AUC, calibration, Hosmer–Lemeshow test, or justify why these were not assessed.*

Response: We have added the AUC as a measure of model discrimination and clarified its assessment in the Statistical Analyses section. The model demonstrated moderate discrimination across the imputed datasets (Methods, Lines 189-191; Results, Lines 219-221).

*6. Ethics and data availability: Please ensure the ethics statement and data availability statement are consistent across the manuscript and submission system. The IRB approval should be stated definitively, including the approving IRB and approval number if available.*

Response: We have included the protocol number in this study (Line 107) and ensured consistency across manuscript and submission system.

*7. Generalizability: Please further temper claims about generalizability, given the single-site VA setting and predominantly male sample. The limitations for non-VA, community-based, female, or more diverse populations should be clearly stated.*

Response: A line has been added further emphasizing the limited generalizability of our conclusions (Line 274-275).

**Minor comments**

*8.Please clarify the timing and funding source of financial incentives.*

Response: The timeline and source of funding for participants is stated in lines 104-105.

*9.Ensure inclusion and exclusion criteria are both clearly listed.*

Response: Inclusion and exclusion criteria are stated more plainly (Line 121-126).

*10. Check the abstract for consistency with the adjusted results, especially age and race/ethnicity findings.*

Response: The abstract is now consistent with the adjusted results. Older age was associated with lower enrollment and Hispanic race/ethnicity experienced greater enrollment as compared to White patients (Line 41-44).

*11. Because no multiple-comparison adjustment was performed, use exploratory language throughout.*

Response: We have revised the manuscript throughout to use more cautious, exploratory language when describing and interpreting the findings. (Abstract, Lines 36-46; Discussion, Lines 253, 283, 308-310).

*12. Please use precise statistical wording, e.g., “All tests were two-sided, and p < 0.05 was considered statistically significant. P-values were not adjusted for multiple comparisons.”*

Response: We have revised the Statistical Analyses section to the suggested wording (Methods, Lines 192-194).

*13. In the response letter, include line numbers and avoid defensive wording such as “I believe.”*

Response: We have avoided the use of defensive wording in our response letter and have provided line numbers when appropriate.

**Reviewer #4:** Table 2 (Crude analyses):

*Please clarify whether Table 2 crude analyses represent (i) univariable logistic regression results, or (ii) LASSO-based variable selection/stability results across imputed datasets. If the latter is intended, this should be clearly relabeled and described in the statistical analyses section to avoid misinterpretation. If univariable logistic regression was performed, pooled estimates under Rubin’s Rules should be reported consistently for all variables.*

**Response:** We have clarified that the crude estimates represent univariable logistic regression analyses rather than LASSO-based variable selection results. Crude estimates for all variables were pooled using Rubin’s Rules (Methods, Lines 187-189; Table 2).
