# Supplementary material for: An analysis of enrollment rates by admitting service for a secondary analysis of enrollment data from a randomized smoking-cessation trial (FIESTA)
Source: PLoS One. 2026 Nov 4;21(11):e0345267. doi: 10.1371/journal.pone.0345267 (PMC13734444; doi:10.1371/journal.pone.0345267)
Supplement: Peer review history

# Peer review: Decision Letter 0

**DOI:** 10.1371/journal.pone.0345267.r001

**Authors:** Jennifer Tucker

25 Sep 2025

Dear Dr. Sherman,

Could you please carefully revise the manuscript to address all comments raised?

Please submit your revised manuscript by Nov 09 2025 11:59PM. If you will need more time than this to complete your revisions, please reply to this message or contact the journal office at plosone@plos.org. When you're ready to submit your revision, log on to https://www.editorialmanager.com/pone/ and select the 'Submissions Needing Revision' folder to locate your manuscript file.

- A rebuttal letter that responds to each point raised by the academic editor and reviewer(s). You should upload this letter as a separate file labeled 'Response to Reviewers'.
- A marked-up copy of your manuscript that highlights changes made to the original version. You should upload this as a separate file labeled 'Revised Manuscript with Track Changes'.
- An unmarked version of your revised paper without tracked changes. You should upload this as a separate file labeled 'Manuscript'.

If you would like to make changes to your financial disclosure, please include your updated statement in your cover letter. Guidelines for resubmitting your figure files are available below the reviewer comments at the end of this letter.

If applicable, we recommend that you deposit your laboratory protocols in protocols.io to enhance the reproducibility of your results. Protocols.io assigns your protocol its own identifier (DOI) so that it can be cited independently in the future. For instructions see: https://journals.plos.org/plosone/s/submission-guidelines#loc-laboratory-protocols. Additionally, PLOS ONE offers an option for publishing peer-reviewed Lab Protocol articles, which describe protocols hosted on protocols.io. Read more information on sharing protocols at https://plos.org/protocols?utm_medium=editorial-email&utm_source=authorletters&utm_campaign=protocols.

We look forward to receiving your revised manuscript.

Kind regards,

Jennifer Tucker, PhD

Staff Editor

PLOS ONE

Journal Requirements:

When submitting your revision, we need you to address these additional requirements.

1. Please ensure that your manuscript meets PLOS ONE's style requirements, including those for file naming. The PLOS ONE style templates can be found at https://journals.plos.org/plosone/s/file?id=wjVg/PLOSOne_formatting_sample_main_body.pdf and https://journals.plos.org/plosone/s/file?id=ba62/PLOSOne_formatting_sample_title_authors_affiliations.pdf

2. Thank you for stating the following in the Acknowledgments Section of your manuscript:

We would like to acknowledge the Robert Wood Johnson Foundation for funding the original 229 FIESTA study (Grant 74140) (JAL,SES).

We note that you have provided funding information that is not currently declared in your Funding Statement. However, funding information should not appear in the Acknowledgments section or other areas of your manuscript. We will only publish funding information present in the Funding Statement section of the online submission form.

Please remove any funding-related text from the manuscript and let us know how you would like to update your Funding Statement. Currently, your Funding Statement reads as follows:

The Robert Wood Johnson Foundation funded the original FIESTA study (Grant 74140) (JAL,SES). No additional funds were provided for this secondary analysis.

Please include your amended statements within your cover letter; we will change the online submission form on your behalf.

3. Please include captions for your Supporting Information files at the end of your manuscript, and update any in-text citations to match accordingly. Please see our Supporting Information guidelines for more information: http://journals.plos.org/plosone/s/supporting-information.

4. If the reviewer comments include a recommendation to cite specific previously published works, please review and evaluate these publications to determine whether they are relevant and should be cited. There is no requirement to cite these works unless the editor has indicated otherwise.

[Note: HTML markup is below. Please do not edit.]

Reviewers' comments:

Reviewer's Responses to Questions

**Comments to the Author**

1. Is the manuscript technically sound, and do the data support the conclusions?

Reviewer #1: Yes

Reviewer #2: Yes

**********

2. Has the statistical analysis been performed appropriately and rigorously?

Reviewer #1: Yes

Reviewer #2: No

**********

3. Have the authors made all data underlying the findings in their manuscript fully available?

The PLOS Data policy

Reviewer #1: Yes

Reviewer #2: No

**********

4. Is the manuscript presented in an intelligible fashion and written in standard English?

Reviewer #1: Yes

Reviewer #2: Yes

**********

Reviewer #1: I read the paper with an interest. It seems like descriptive study. There is an important finding to point, the higher recruitment rates of patients refered from psychiatry service. On the other hand sone previous studies have pointed that physiciatrist are the least motivated group to give smoking cessation service or teach tobacco related lectures, due to the relaxing effect on, seen in the following article ( DOI: 10.4103/1357-6283.178602, Education for Health • Volume 28 • Issue 3 (December 2015) )‘The two lecturers who were unconvinced that tobacco merited special attention were from the psychiatry and pharmacology departments. While one of two faculty members interviewed from the psychiatry department was in favor of using tobacco as an exemplar for teaching students about the process of addiction, a second psychiatrist was of the opinion that smoking was a useful coping mechanism for patients suffering from conditions such as anxiety, depression, and schizophrenia. ‘

-Line 51:…in 2021,…where?? (in general or worldwide or US)

-Line 65: Please add reference for examples of hospital-based programmes, such as the following recent paper: DOI: 10.18332/tid/191239

-In table 1: ‘Medicine’ as name of department is not clear, which department of medicine, (internal medicine or surgery departments, or pulmonology or cardiology?)

Reviewer #2: This secondary analysis of recruitment data from the FIESTA RCT addresses a practical but under-studied question: does admitting service (Medicine vs. Psychiatry) influence enrolment in an inpatient smoking-cessation trial? The manuscript is clearly written, the dataset is unusual, and the findings could inform recruitment planning. Nevertheless, methodological transparency and completeness of reporting require attention before the paper reaches PLOS ONE standards.

MAJOR REVISIONS

Study design mis-labelling

– Title page & Abstract, l. 3-4: Replace “a randomized control smoking cessation study” with “a secondary analysis of recruitment data from a randomized smoking-cessation trial (FIESTA).”

– Introduction, l. 46-48: Add explicit sentence: “The present work is a secondary analysis of the FIESTA RCT (NCT02506829).”

CONSORT flow diagram missing

– Insert Figure 1 (after l. 88) showing screened → eligible → approached → enrolled, with reasons for non-enrolment, stratified by Medicine / Psychiatry / Other.

Sparse “Other” admitting service

– Methods, l. 117-119 & Table 2: Only 22 patients (8 %) yield an imprecise aOR 0.71 (95 % CI 0.25–2.04).

– Collapse “Other” into “Medicine” or exclude and supply sensitivity analysis (supplement or appendix).

Multiple-imputation reporting incomplete

– Methods, l. 137-143: State: (a) m = 20 imputed datasets, (b) 50 burn-in iterations, (c) predictor list (age, sex, race, education, cigarettes/day, service, LOS), (d) fraction of missing information (FMI) for each coefficient.

– Clarify why education “Missing” (n = 40, Table 1) is excluded from adjusted model (l. 148).

Confounding control

– Age and length-of-stay (LOS) are available in FIESTA baseline files but not used. Add both covariates to the final model or explain their absence (Discussion, l. 190-192).

Multiplicity of tests

– Footnote to Table 2: Apply Holm–Bonferroni correction across 20 pairwise tests; report adjusted p-values or label comparison “exploratory.”

Data-availability statement

– l. 245-247: Replace current sentence with:

“De-identified participant data are available from the VA New York Harbor Healthcare System for researchers who obtain the necessary VA data-use agreement (contact scott.sherman@nyulangone.org).”

MINOR REVISIONS

Abstract results, l. 33-35: Add 95 % CIs for Psychiatry vs Medicine (3.20, 1.63–6.26) and high-school vs college (0.19, 0.07–0.51).

Table 1 footnote, l. 154: Use χ² for cells ≥5; Fisher exact only for cells <5; be consistent.

Language, l. 198: Change “make studies generalizable” to “may improve representativeness.”

References, l. 250: Update CDC smoking prevalence to 2023 NHIS (11.5 % adults).

Supporting Information: Upload (a) CONSORT flow diagram, (b) R script (LASSO + mice code), (c) sensitivity analysis without “Other” service.

**********

what does this mean?). If published, this will include your full peer review and any attached files.

If you choose “no”, your identity will remain anonymous but your review may still be made public.

**Do you want your identity to be public for this peer review?**  For information about this choice, including consent withdrawal, please see our Privacy Policy

Reviewer #1: No

Reviewer #2: **Yes:**  Wan Jia Aaron He

**********

[NOTE: If reviewer comments were submitted as an attachment file, they will be attached to this email and accessible via the submission site. Please log into your account, locate the manuscript record, and check for the action link "View Attachments". If this link does not appear, there are no attachment files.]

While revising your submission, please upload your figure files to the Preflight Analysis and Conversion Engine (PACE) digital diagnostic tool, https://pacev2.apexcovantage.com/. PACE helps ensure that figures meet PLOS requirements. To use PACE, you must first register as a user. Registration is free. Then, login and navigate to the UPLOAD tab, where you will find detailed instructions on how to use the tool. If you encounter any issues or have any questions when using PACE, please email PLOS at figures@plos.org. Please note that Supporting Information files do not need this step.

# Author comment: Author response to Decision Letter 1

**DOI:** 10.1371/journal.pone.0345267.r002

21 Nov 2025

Reviewer #1: I read the paper with an interest. It seems like descriptive study. There is an important finding to point, the higher recruitment rates of patients refered from psychiatry service. On the other hand sone previous studies have pointed that physiciatrist are the least motivated group to give smoking cessation service or teach tobacco related lectures, due to the relaxing effect on, seen in the following article ( DOI: 10.4103/1357-6283.178602, Education for Health • Volume 28 • Issue 3 (December 2015) )‘The two lecturers who were unconvinced that tobacco merited special attention were from the psychiatry and pharmacology departments. While one of two faculty members interviewed from the psychiatry department was in favor of using tobacco as an exemplar for teaching students about the process of addiction, a second psychiatrist was of the opinion that smoking was a useful coping mechanism for patients suffering from conditions such as anxiety, depression, and schizophrenia. ‘

Response: Thank you for this thoughtful feedback. We are grateful for your interest in our work and have addressed your comments below.

-Line 51:…in 2021,…where?? (in general or worldwide or US)

Response: We have updated the data to be from the 2023 NHIS publication as suggested by the second reviewer, and have clarified that the data refers to US smoking rates.

-Line 65: Please add reference for examples of hospital-based programmes, such as the following recent paper: DOI: 10.18332/tid/191239

Response: Thank you for this suggestion, I have included this interesting study as an example of a hospital-based smoking cessation program

-In table 1: ‘Medicine’ as name of department is not clear, which department of medicine, (internal medicine or surgery departments, or pulmonology or cardiology?)

Response: I have clarified that Medicine refers to Internal Medicine, both in the tables and as it appears in the results and discussion sections.

Reviewer #2: This secondary analysis of recruitment data from the FIESTA RCT addresses a practical but under-studied question: does admitting service (Medicine vs. Psychiatry) influence enrolment in an inpatient smoking-cessation trial? The manuscript is clearly written, the dataset is unusual, and the findings could inform recruitment planning. Nevertheless, methodological transparency and completeness of reporting require attention before the paper reaches PLOS ONE standards.

MAJOR REVISIONS

Study design mis-labelling

– Title page & Abstract, l. 3-4: Replace “a randomized control smoking cessation study” with “a secondary analysis of recruitment data from a randomized smoking-cessation trial (FIESTA).”

Response: Thank you. I have updated the title as suggested to more clearly represent the paper

– Introduction, l. 46-48: Add explicit sentence: “The present work is a secondary analysis of the FIESTA RCT (NCT02506829).”

Response: Thank you. I have added the suggest line to the abstract

CONSORT flow diagram missing

– Insert Figure 1 (after l. 88) showing screened → eligible → approached → enrolled, with reasons for non-enrolment, stratified by Medicine / Psychiatry / Other.

Response: Thank you for this helpful suggestion. We added a CONSORT flow diagram (Supplementary Figure 1) summarizing the numbers screened, eligible, approached, and enrolled, with reasons for non-enrollment, stratified by Medicine, Psychiatry, and other services.

Sparse “Other” admitting service

– Methods, l. 117-119 & Table 2: Only 22 patients (8 %) yield an imprecise aOR 0.71 (95 % CI 0.25–2.04).

– Collapse “Other” into “Medicine” or exclude and supply sensitivity analysis (supplement or appendix).

Response: Thank you for pointing this out. We collapsed the “Other” admitting service (n = 22) with “Medicine” in all analyses to improve model stability and clarity. This change is reflected in the Methods, Table 1, and Table 2. Significance remained unchanged when excluding the patients from “Other” department in the sensitivity analysis (see Supplementary Table 2).

Multiple-imputation reporting incomplete

– Methods, l. 137-143: State: (a) m = 20 imputed datasets, (b) 50 burn-in iterations, (c) predictor list (age, sex, race, education, cigarettes/day, service, LOS), (d) fraction of missing information (FMI) for each coefficient.

Response: Thank you for this important feedback. We have added the requested items to the methods section as bellows:

“Twenty imputed datasets were generated, each based on 50 iterations. For each imputed dataset, variable selection was carried out using Least Absolute Shrinkage and Selection Operator (LASSO) approach to build a predictive model for the primary analysis.”

“The other predictors included sex, age at baseline, race/ethnicity, education, and number of cigarettes per day.”

“Fractions of missing information were also computed for each coefficient to quantify the loss of stability attributable to missing data.”

– Clarify why education “Missing” (n = 40, Table 1) is excluded from adjusted model (l. 148).

Response: Thank you for noting this. For education, race/ethnicity and smoking history “Missing” values were imputed before fitting the model and therefore were not included as visible category in the adjusted model.

Confounding control

– Age and length-of-stay (LOS) are available in FIESTA baseline files but not used. Add both covariates to the final model or explain their absence (Discussion, l. 190-192).

Response: Thank you for this important feedback. We revised the entire analysis by adding Age variable. However, LOS was only found available among the patients who were enrolled in the study. Since our study compared enrolled versus non-enrolled, LOS could not be included.

Multiplicity of tests

– Footnote to Table 2: Apply Holm–Bonferroni correction across 20 pairwise tests; report adjusted p-values or label comparison “exploratory.”

Response: Thank you for this thoughtful suggestion. Since our study is a secondary analysis of recruitment rates, we aimed to present the effect sizes rather than testing pre-specified hypotheses, we did not apply family-wise error procedures. We have labeled all comparisons as exploratory and interpreted cautiously in the footnote of Table 2 as “P-values are unadjusted and interpreted as exploratory given the secondary nature of this analysis”.

Data-availability statement

– l. 245-247: Replace current sentence with:

“De-identified participant data are available from the VA New York Harbor Healthcare System for researchers who obtain the necessary VA data-use agreement (contactscott.sherman@nyulangone.org).”

Response: Thank you for this suggestion. The lines suggested in this comment are within the references so I have added the suggested statement to the Study Design section of methods instead.

MINOR REVISIONS

Abstract results, l. 33-35: Add 95 % CIs for Psychiatry vs Medicine (3.20, 1.63–6.26) and high-school vs college (0.19, 0.07–0.51).

Response: Thank you for this suggestion, confidence intervals have been added to the abstract results.

Table 1 footnote, l. 154: Use χ² for cells ≥5; Fisher exact only for cells <5; be consistent.

Response: This has been addressed in the captions for supplemental figures and the footnote has been taken out.

Language, l. 198: Change “make studies generalizable” to “may improve representativeness.”

Response: Thank you for this suggestion. We hope to keep the wording to generalization rather than representation. Our aim is to make sure smoking-cessation studies are applicable to the general public, not to sway representation in studies to certain demographics.

References, l. 250: Update CDC smoking prevalence to 2023 NHIS (11.5 % adults).

Response: I have updated the source to one from the Office of Disease Prevention and Health Promotion that utilizes the NHIS 11.5% statistic for a more updated report than the 2021 CDC one I have previously included.

Supporting Information: Upload (a) CONSORT flow diagram, (b) R script (LASSO + mice code), (c) sensitivity analysis without “Other” service.

Response: The requested supporting information has been added.

Attachment Submitted filename: Response letter - 11-10-2025.docx

# Peer review: Decision Letter 1

**DOI:** 10.1371/journal.pone.0345267.r003

**Authors:** Jennifer Tucker

13 Apr 2026

Dear Dr. Sherman,

Thank you for submitting your manuscript to PLOS ONE. After careful consideration, we feel that it has merit but does not fully meet PLOS ONE’s publication criteria as it currently stands. Therefore, we invite you to submit a revised version of the manuscript that addresses the points raised during the review process.

Please submit your revised manuscript by May 28 2026 11:59PM. If you will need more time than this to complete your revisions, please reply to this message or contact the journal office at plosone@plos.org. When you're ready to submit your revision, log on to https://www.editorialmanager.com/pone/ and select the 'Submissions Needing Revision' folder to locate your manuscript file.

- A letter that responds to each point raised by the academic editor and reviewer(s). You should upload this letter as a separate file labeled 'Response to Reviewers'.
- A marked-up copy of your manuscript that highlights changes made to the original version. You should upload this as a separate file labeled 'Revised Manuscript with Track Changes'.
- An unmarked version of your revised paper without tracked changes. You should upload this as a separate file labeled 'Manuscript'.

If you would like to make changes to your financial disclosure, please include your updated statement in your cover letter. Guidelines for resubmitting your figure files are available below the reviewer comments at the end of this letter.

If applicable, we recommend that you deposit your laboratory protocols in protocols.io to enhance the reproducibility of your results. Protocols.io assigns your protocol its own identifier (DOI) so that it can be cited independently in the future. For instructions see: https://journals.plos.org/plosone/s/submission-guidelines#loc-laboratory-protocols. Additionally, PLOS ONE offers an option for publishing peer-reviewed Lab Protocol articles, which describe protocols hosted on protocols.io. Read more information on sharing protocols at https://plos.org/protocols?utm_medium=editorial-email&utm_source=authorletters&utm_campaign=protocols.

As the corresponding author, your ORCID iD is verified in the submission system and will appear in the published article. PLOS supports the use of ORCID, and we encourage all coauthors to register for an ORCID iD and use it as well. Please encourage your coauthors to verify their ORCID iD within the submission system before final acceptance, as unverified ORCID iDs will not appear in the published article. *Only*  the individual author can complete the verification step; PLOS staff *cannot*  verify ORCID iDs on behalf of authors.

We look forward to receiving your revised manuscript.

Kind regards,

Jennifer Tucker, PhD

Staff Editor

PLOS One

Journal Requirements:

If the reviewer comments include a recommendation to cite specific previously published works, please review and evaluate these publications to determine whether they are relevant and should be cited. There is no requirement to cite these works unless the editor has indicated otherwise.

[Note: HTML markup is below. Please do not edit.]

Reviewers' comments:

Reviewer's Responses to Questions

**Comments to the Author**

Reviewer #1: All comments have been addressed

Reviewer #2: All comments have been addressed

Reviewer #3: All comments have been addressed

Reviewer #4: (No Response)

**********

2. Is the manuscript technically sound, and do the data support the conclusions?

Reviewer #1: Yes

Reviewer #2: Yes

Reviewer #3: Yes

Reviewer #4: Partly

**********

3. Has the statistical analysis been performed appropriately and rigorously?

Reviewer #1: Yes

Reviewer #2: Yes

Reviewer #3: Yes

Reviewer #4: No

**********

4. Have the authors made all data underlying the findings in their manuscript fully available?

The PLOS Data policy

Reviewer #1: Yes

Reviewer #2: Yes

Reviewer #3: Yes

Reviewer #4: Yes

**********

5. Is the manuscript presented in an intelligible fashion and written in standard English?

Reviewer #1: Yes

Reviewer #2: Yes

Reviewer #3: Yes

Reviewer #4: Yes

**********

Reviewer #1: (No Response)

Reviewer #2: Thank you for the careful revision and detailed response to the previous round of review. The manuscript has improved substantially, and many of the earlier concerns have been addressed. In particular, I appreciate the clarification of the study title, the handling of the sparse “Other” admitting service category, the inclusion of age in the adjusted analysis, the expanded reporting of the imputation approach, and the addition of supporting materials.

My comments are as follows:

1. Please state explicitly in the Introduction that this manuscript is a secondary analysis. The Abstract now notes that this is a secondary analysis of the FIESTA RCT, which is helpful. However, this should also be stated clearly in the Introduction so that the study design is explicit at the outset of the main text. A brief sentence identifying this work as a secondary analysis of recruitment data from the FIESTA trial (NCT02506829) would address this.

2. Please reconcile the race/ethnicity findings across the Abstract, Results, Discussion, and Table 2. At present, the Abstract states that race did not significantly impact recruitment. However, Table 2 reports a statistically significant association for Hispanic participants compared with non-Hispanic White participants, and the Results/Discussion also describe higher recruitment among Hispanic patients. These statements should be made consistent throughout the manuscript. If the intent is to emphasize the exploratory nature of the subgroup comparisons, that should be stated clearly, but the wording should still accurately reflect the reported estimates.

3. Please align the stated objective with the analyses presented. The Introduction states that the primary objective was to determine whether recruitment and retention were higher on the Medical Service than on the Psychiatry Service. However, the manuscript as currently written appears to analyze recruitment/enrollment only. Please revise the objective language accordingly, unless retention analyses are also intended to be included.

4. Please clarify the Data Availability Statement and ensure consistency across submission materials. The manuscript text states that de-identified participant data are available through the VA system with the appropriate data-use agreement, whereas the submission materials indicate that all relevant data are within the manuscript and Supporting Information files. Please provide one clear and accurate Data Availability Statement that reflects exactly what is publicly available and what, if anything, remains under restricted access.

5. Please moderate the language regarding generalizability. The manuscript makes an important contribution by showing successful recruitment of groups often underrepresented in clinical research. However, some statements about making studies more “generalizable to the smoking population” remain stronger than warranted for a single-site VA study with a predominantly male sample. I suggest slightly tempering this language and distinguishing more clearly between inclusiveness within this study context and broader external validity.

6. Please revise the Discussion for consistency regarding which variables were available among screened patients. The Discussion states that, among screened patients, the authors only had access to admitting service, sex, race, education, and smoking history. However, age was included in the adjusted model and reported in the Results. Please revise this description so it is consistent with the variables actually used in the analysis.

7. Abstract Methods: please include age among the predictors. The Abstract Methods currently list admitting service, sex, race/ethnicity, education, and cigarettes per day, but age was also included in the adjusted model and was statistically significant. Please add age to the Abstract Methods for completeness and consistency.

8. Please cite the recruitment flow diagram explicitly in the main text. The addition of the recruitment/CONSORT-style flow diagram is appreciated. Please ensure that it is explicitly referenced in the main text so that readers can readily identify it.

9. Please ensure that the ethics information is consistent across the manuscript and submission system. The manuscript indicates that the protocol received institutional review board approval, whereas the submission metadata appears to list the ethics statement as “N/A.” Please verify and correct this so that the ethics information is consistent across all submission components.

10. Please conduct a final editorial consistency check. Before final acceptance, I recommend a final review for consistency in terminology, formatting, and numerical reporting across the manuscript, tables, and supplementary materials.

Reviewer #3: (No Response)

Reviewer #4: The manuscript requires further improvement.

Line 30: The term secondary analyses is to be used.

Line 87: Allocation concealment and blinding are to be described.

Line 89: When are the financial incentives provided, at each visit or at the end of the study? Are these funded through research grants?

Line 117: The exclusion criteria is to be added.

The percentage of missing data to be stated along with the missing data pattern.

Line 140-141: The sentence requires revision e.g. After LASSO-based variable selection, regression models were fitted, and the estimates were pooled along with 95% confidence intervals (CIs) and p-values using Rubin’s Rules.

Line 127: The word Analysis to be replaced with Statistical Analyses.

The statistical tests employed in the results section are to be clearly stated in the statistical analyses section.

Line 156: Multivariable results? The sentence requires revision.

Table 1: Where applicable, percentage figure is to be included.

Line 171: The coding variable for the recruitment (outcome variable) is to be specified.

Tables 1 and 2: For smoking history/cigarettes per day respectively, the categories overlap (e.g., someone who smokes 4 cigarettes fits both <5 and <10). It should be mutually exclusive. Smoking can be categorized into mutually exclusive groups (0–5, 6–10, 11–20, >20 cigarettes/day), with 0–5 cigarettes/day as the reference category as highligted in the suppementary tables. Symbol * is to be clearly denoted in the table footnote.

Table 2: Crude (unadjusted) analyses are to be presented alongside adjusted analyses. The model selection strategy, handling of confounders (selection criteria), and assessment of model fit are to be described. Column size for FMI could be reduced.

Line 177: To state P value (unadjusted for multiple comparisons).

A statement ‘No adjustments were made for multiple comparisons due to the exploratory nature of the study’ to be included in the method section.

The fulfillment of the statistical assumptions, such as multicollinearity, etc., is to be stated.

Supplementary Table 1: symbol <= is to be replaced with ≤.

Supplementray Table 1 and Supplementary Table 2: If the P values here are unadjusted for multiple comparisons, it is to be clearly stated in the table footnote.

The primary objective ‘determine whether rates of FIESTA participant recruitment and retention were higher on the Medical Service than on the Psychiatry Service.’ is leaning towards hypothesis testing. While multiple comparisons were performed and since no adjustment was applied, this may increase the risk of Type I error and should be acknowledged or justified. If the study is an exploratory nature, it should be stated clearly and reflected in the manuscript e.g. introduction, method, discussion.

Some references did not conform to the journal’s formatting requirements.

**********

what does this mean?). If published, this will include your full peer review and any attached files.

If you choose “no”, your identity will remain anonymous but your review may still be made public.

**Do you want your identity to be public for this peer review?**  For information about this choice, including consent withdrawal, please see our Privacy Policy

Reviewer #1: No

Reviewer #2: No

Reviewer #3: No

Reviewer #4: No

**********

[NOTE: If reviewer comments were submitted as an attachment file, they will be attached to this email and accessible via the submission site. Please log into your account, locate the manuscript record, and check for the action link "View Attachments". If this link does not appear, there are no attachment files.]

To ensure your figures meet our technical requirements, please review our figure guidelines: https://journals.plos.org/plosone/s/figures

You may also use PLOS’s free figure tool, NAAS, to help you prepare publication quality figures: https://journals.plos.org/plosone/s/figures#loc-tools-for-figure-preparation.

NAAS will assess whether your figures meet our technical requirements by comparing each figure against our figure specifications.

# Author comment: Author response to Decision Letter 2

**DOI:** 10.1371/journal.pone.0345267.r004

12 Jun 2026

Ref: PONE-D-25-20584

Dear Dr. Jennifer Tucker,

Thank you for extending the opportunity to revise our manuscript. We appreciate your comments and have carefully considered your feedback and made revisions accordingly. We have provided a comprehensive response addressing all the reviews and incorporated both reviewers’ feedback into our revision.

This paper has not been submitted or published elsewhere. All authors approved the final manuscript. There are no conflicts of interest. We appreciate your interest in our work.

Sincerely,

Kayla Koffler, MSIII

Reviewer #2:

1. Please state explicitly in the Introduction that this manuscript is a secondary analysis. The Abstract now notes that this is a secondary analysis of the FIESTA RCT, which is helpful. However, this should also be stated clearly in the Introduction so that the study design is explicit at the outset of the main text. A brief sentence identifying this work as a secondary analysis of recruitment data from the FIESTA trial (NCT02506829) would address this.

Response: Thank you for this feedback, we have added this into the introduction (lines 87-88)

2. Please reconcile the race/ethnicity findings across the Abstract, Results, Discussion, and Table 2. At present, the Abstract states that race did not significantly impact recruitment. However, Table 2 reports a statistically significant association for Hispanic participants compared with non-Hispanic White participants, and the Results/Discussion also describe higher recruitment among Hispanic patients. These statements should be made consistent throughout the manuscript. If the intent is to emphasize the exploratory nature of the subgroup comparisons, that should be stated clearly, but the wording should still accurately reflect the reported estimates.

Response: Thank you for this important observation. We have revised the inconsistencies and made statements aligning with the table results throughout the manuscript. [MH1.1]

3. Please align the stated objective with the analyses presented. The Introduction states that the primary objective was to determine whether recruitment and retention were higher on the Medical Service than on the Psychiatry Service. However, the manuscript as currently written appears to analyze recruitment/enrollment only. Please revise the objective language accordingly, unless retention analyses are also intended to be included.

Response: Thank you for this suggestion. We revised the primary objective language to focus on recruitment as retention analyses were not performed in the present manuscript.

4. Please clarify the Data Availability Statement and ensure consistency across submission materials. The manuscript text states that de-identified participant data are available through the VA system with the appropriate data-use agreement, whereas the submission materials indicate that all relevant data are within the manuscript and Supporting Information files. Please provide one clear and accurate Data Availability Statement that reflects exactly what is publicly available and what, if anything, remains under restricted access.

The submission materials are correct – all relevant data are within the manuscript and Supporting Information files.

5. Please moderate the language regarding generalizability. The manuscript makes an important contribution by showing successful recruitment of groups often underrepresented in clinical research. However, some statements about making studies more “generalizable to the smoking population” remain stronger than warranted for a single-site VA study with a predominantly male sample. I suggest slightly tempering this language and distinguishing more clearly between inclusiveness within this study context and broader external validity.

Response: I believe that the language here is emphasizing the importance of the goal of making smoking cessation trials more generalizable to the public, not stating that this paper will facilitate that generalizability. I added in a line more clearly stating the limits of this study in that mission (lines 275-277)

6. Please revise the Discussion for consistency regarding which variables were available among screened patients. The Discussion states that, among screened patients, the authors only had access to admitting service, sex, race, education, and smoking history. However, age was included in the adjusted model and reported in the Results. Please revise this description so it is consistent with the variables actually used in the analysis.

Response: We revised the description in the discussion to include age as a variable we had access to (line 295)

7. Abstract Methods: please include age among the predictors. The Abstract Methods currently list admitting service, sex, race/ethnicity, education, and cigarettes per day, but age was also included in the adjusted model and was statistically significant. Please add age to the Abstract Methods for completeness and consistency.

Response: We appreciate the important observation. We added age to the Abstract Methods to ensure completeness and consistency with the primary analyses.

8. Please cite the recruitment flow diagram explicitly in the main text. The addition of the recruitment/CONSORT-style flow diagram is appreciated. Please ensure that it is explicitly referenced in the main text so that readers can readily identify it.

Response: Thank you for the suggestion. We included a citation of “Participant screening, eligibility assessment, and enrollment are summarized in Supplementary Figure 1.” in the Results section within the main text of the manuscript. We also added “Overall, 929 patients were screened, 268 met eligibility criteria, and 182 were randomized” in the eligibility criteria section to briefly describe the recruitment flow.

9. Please ensure that the ethics information is consistent across the manuscript and submission system. The manuscript indicates that the protocol received institutional review board approval, whereas the submission metadata appears to list the ethics statement as “N/A.” Please verify and correct this so that the ethics information is consistent across all submission components.

The FIESTA trial received IRB approval, which would cover these analyses. So, the correct answer should be that it was approved (rather than “N/A”).

10. Please conduct a final editorial consistency check. Before final acceptance, I recommend a final review for consistency in terminology, formatting, and numerical reporting across the manuscript, tables, and supplementary materials.

Response: Thank you for this suggestion, we have conducted several final read throughs for consistency.

Reviewer #4:

Line 30: The term secondary analyses is to be used.

Response: Updated the terminology to read secondary analysis.

Line 87: Allocation concealment and blinding are to be described.

Response: Thank you for the helpful suggestion. We added “Participants and study staff were not blinded to treatment assignment” to the main text within the manuscript to clarify the allocation concealment and blinding for the study.

Line 89: When are the financial incentives provided, at each visit or at the end of the study? Are these funded through research grants?

Response: We have added a clarification to the timing and funding (104-105)

Line 117: The exclusion criteria is to be added.

Response: Exclusion criteria has been added.

The percentage of missing data to be stated along with the missing data pattern.

Response: Thank you for the comments. We added “Missingness ranged from 3.7% to 14.9% across all covariates, with the highest missingness observed for education and smoking history variables” along the missing data pattern description in the analysis section.

Line 140-141: The sentence requires revision e.g. After LASSO-based variable selection, regression models were fitted, and the estimates were pooled along with 95% confidence intervals (CIs) and p-values using Rubin’s Rules.

Response: Thank you for the comment. The sentence was revised for clarity now stated “After LASSO-based variable selection, regression models were fitted, and the estimates were pooled along with 95% confidence intervals (CIs) and p-values using Rubin’s Rules” in the Statistical Analyses section.

Line 127: The word Analysis to be replaced with Statistical Analyses.

Response: Thank you for the suggestion. The heading was replaced with Statistical Analyses for clarity purpose.

The statistical tests employed in the results section are to be clearly stated in the statistical analyses section.

Response: Thank you for this important note. We included “Baseline age was compared between groups using a two-sample t-test. Categorical variables were compared using χ² tests or Fisher exact tests when expected cell counts < 5” to describe the descriptive analyses. For the inferential analyses, LASSO approach and multivariable logistic regression were addressed in the Statistical Analyses section as suggested in the previous comments.

Line 156: Multivariable results? The sentence requires revision.

Response: We appreciate this important observation. The sentence was revised to “Adjusted multivariable logistic regression results are presented in Table 2” for completeness.

Table 1: Where applicable, percentage figure is to be included.

Response: Thank you for this helpful comment. Column percentages were added to Table 1 to provide comparability that raw frequencies alone cannot offer.

Line 171: The coding variable for the recruitment (outcome variable) is to be specified.

Response: Thank you for the observation. A statement of “The outcome was coded as a binary outcome variable (0 = did not participate, 1 = participated)” was added to both Methods section and footnote in Table 2.

Tables 1 and 2: For smoking history/cigarettes per day respectively, the categories overlap (e.g., someone who smokes 4 cigarettes fits both <5 and <10). It should be mutually exclusive. Smoking can be categorized into mutually exclusive groups (0–5, 6–10, 11–20, >20 cigarettes/day), with 0–5 cigarettes/day as the reference category as highlighted in the supplementary tables. Symbol * is to be clearly denoted in the table footnote.

Response: Thank you for the comment. The categories under cigarettes per day variable were mutually exclusive throughout the analyses. The labels of categories were revised to ≤ 5, >5 and ≤ 10, > 10 and ≤ 20, and > 20 for less confusion. The Symbol * has been removed from both Table 1 and Table 2.

Table 2: Crude (unadjusted) analyses are to be presented alongside adjusted analyses. The model selection strategy, handling of confounders (selection criteria), and assessment of model fit are to be described. Column size for FMI could be reduced.

Response: We appreciate this comment. Crude odds ratio was added to Table 2 along with a p-value. The Statistical Analyses was further revised to “Missingness ranged from 3.7% to 14.9% across all covariates, with the highest missingness observed for education and smoking history variables. Missing data was imputed using multiple imputations by chained equations (MICE). Twenty imputed datasets were generated, each based on 50 iterations. For each imputed dataset, variable selection was carried out using Least Absolute Shrinkage and Selection Operator (LASSO) approach to build a predictive model for the primary analysis. Variables consistently retained across all imputed datasets were included in the final multivariable logistic regression model. Demographic and smoking-related variables were considered as potential confounders based on clinical relevance. After LASSO-based variable selection, regression models were fitted, and the estimates were pooled along with 95% confidence intervals (CIs) and p-values using Rubin’s Rules. Fractions of missing information were also computed for each coefficient to quantify the loss of stability attributable to missing data. No adjustments were made for multiple comparisons due to the exploratory nature of the study. All statistics tests were 2-sided with p-value < 0.05 testing significance. Analyses were performed with R studio, version 2023.06.2. MICE R packages were used to inspect and impute datasets” for more details to describe model selection, handling of confounders and assessment of model. The formatting of the FMI column in Table 2 was adjusted as suggested.

Line 177: To state P value (unadjusted for multiple comparisons).

Response: Thank you for the comment. “P-values are unadjusted and interpreted as exploratory given the secondary nature of this analysis” was added to the footnote in Table 2.

A statement ‘No adjustments were made for multiple comparisons due to the exploratory nature of the study’ to be included in the method section.

Response: Thank you for the important suggestion. This statement was added to the Statistical Analyses section in the Methods.

The fulfillment of the statistical assumptions, such as multicollinearity, etc., is to be stated.

Response: Thank you for the comment. We added “Multicollinearity was assessed prior to model fitting using variance inflation factors, and multicollinearity was not identified” to address the fulfilment of the statistical assumption before the final model estimation.

Supplementary Table 1: symbol <= is to be replaced with ≤.

Response: Thank you for this helpful comment. The symbol was corrected throughout the main manuscript as well as the supplementary materials.

Supplementary Table 1 and Supplementary Table 2: If the P values here are unadjusted for multiple comparisons, it is to be clearly stated in the table footnote.

Response: Thank you for this important observation. Footnotes in all supplementary tables were revised to clarify that p-values were unadjusted for multiple comparisons.

The primary objective ‘determine whether rates of FIESTA participant recruitment and retention were higher on the Medical Service than on the Psychiatry Service.’ is leaning towards hypothesis testing. While multiple comparisons were performed and since no adjustment was applied, this may increase the risk of Type I error and should be acknowledged or justified. If the study is an exploratory nature, it should be stated clearly and reflected in the manuscript e.g. introduction, method, discussion.

Response: Thank you for this observation, I have made note of this Type I error risk in the introduction (lines 87-90) and discussion (lines 302-304).

Some references did not conform to the journal’s formatting requirements.

Response: Thank you for pointing this out, we have updated the references to better fit with NLM/ICMJE style formatting.

Attachment Submitted filename: plosone response letter.docx

# Peer review: Decision Letter 2

**DOI:** 10.1371/journal.pone.0345267.r005

**Authors:** Emma Campbell

17 Jul 2026

Dear Dr. Sherman,

Thank you for submitting your manuscript to PLOS ONE. After careful consideration, we feel that it has merit but does not fully meet PLOS ONE’s publication criteria as it currently stands. Therefore, we invite you to submit a revised version of the manuscript that addresses the points raised during the review process.

**The revised manuscript has been assessed by the reviewers and their comments can be found below. Please ensure you fully review the reports and make all of the required revisions to address the concerns raised.**

Please submit your revised manuscript by Aug 31 2026 11:59PM. If you will need more time than this to complete your revisions, please reply to this message or contact the journal office at plosone@plos.org. When you're ready to submit your revision, log on to https://www.editorialmanager.com/pone/ and select the 'Submissions Needing Revision' folder to locate your manuscript file.

- A letter that responds to each point raised by the academic editor and reviewer(s). You should upload this letter as a separate file labeled 'Response to Reviewers'.
- A marked-up copy of your manuscript that highlights changes made to the original version. You should upload this as a separate file labeled 'Revised Manuscript with Track Changes'.
- An unmarked version of your revised paper without tracked changes. You should upload this as a separate file labeled 'Manuscript'.

If you would like to make changes to your financial disclosure, please include your updated statement in your cover letter. Guidelines for resubmitting your figure files are available below the reviewer comments at the end of this letter.

If applicable, we recommend that you deposit your laboratory protocols in protocols.io to enhance the reproducibility of your results. Protocols.io assigns your protocol its own identifier (DOI) so that it can be cited independently in the future. For instructions see: https://journals.plos.org/plosone/s/submission-guidelines#loc-laboratory-protocols. Additionally, PLOS ONE offers an option for publishing peer-reviewed Lab Protocol articles, which describe protocols hosted on protocols.io. Read more information on sharing protocols at https://plos.org/protocols?utm_medium=editorial-email&utm_source=authorletters&utm_campaign=protocols.

As the corresponding author, your ORCID iD is verified in the submission system and will appear in the published article. PLOS supports the use of ORCID, and we encourage all coauthors to register for an ORCID iD and use it as well. Please encourage your coauthors to verify their ORCID iD within the submission system before final acceptance, as unverified ORCID iDs will not appear in the published article. *Only*  the individual author can complete the verification step; PLOS staff *cannot*  verify ORCID iDs on behalf of authors.

We look forward to receiving your revised manuscript.

Kind regards,

Emma Campbell, Ph.D

Staff Editor

PLOS One

**Journal Requirements:**

If the reviewer comments include a recommendation to cite specific previously published works, please review and evaluate these publications to determine whether they are relevant and should be cited. There is no requirement to cite these works unless the editor has indicated otherwise.

[Note: HTML markup is below. Please do not edit.]

Reviewers' comments:

Reviewer's Responses to Questions

**Comments to the Author**

Reviewer #2: All comments have been addressed

Reviewer #4: (No Response)

**********

2. Is the manuscript technically sound, and do the data support the conclusions?

Reviewer #2: Yes

Reviewer #4: Partly

**********

3. Has the statistical analysis been performed appropriately and rigorously?

Reviewer #2: Yes

Reviewer #4: No

**********

4. Have the authors made all data underlying the findings in their manuscript fully available?

The PLOS Data policy

Reviewer #2: Yes

Reviewer #4: Yes

**********

5. Is the manuscript presented in an intelligible fashion and written in standard English?

Reviewer #2: Yes

Reviewer #4: Yes

**********

**Reviewer #2:**  The manuscript has improved after revision, particularly with the clarification that this is a secondary analysis, the revised focus on recruitment, the addition of unadjusted ORs, and correction of the cigarette-use categories. However, several methodological and reporting issues remain and should be addressed before publication in PLOS ONE.

Major comments

1. Allocation concealment: The authors describe lack of blinding, but allocation concealment for the parent FIESTA trial remains unclear. Please briefly describe the randomization/allocation concealment procedure or cite the parent trial protocol/publication. If not relevant to this recruitment analysis, please state this explicitly.

2. Outcome definition: Please clearly define the binary outcome used in the logistic regression. Does “participation” mean consent, enrollment, randomization, or baseline visit attendance? Use the same term consistently throughout the manuscript.

3. Missing data:The manuscript reports the percentage range of missing data, but not the missing-data pattern. Please describe whether missingness was monotone or non-monotone, whether variables were commonly missing together, and why MICE was considered appropriate.

4. Model selection and confounding: The LASSO variable-selection procedure needs clearer description. Please specify candidate variables, the threshold for being “consistently retained” across imputed datasets, and how clinically important confounders were handled if not selected by LASSO.

5. Model fit: VIF and fraction of missing information do not assess logistic regression model fit. Please report appropriate model performance/fit measures, such as C-statistic/AUC, calibration, Hosmer–Lemeshow test, or justify why these were not assessed.

6. Ethics and data availability:Please ensure the ethics statement and data availability statement are consistent across the manuscript and submission system. The IRB approval should be stated definitively, including the approving IRB and approval number if available.

7. Generalizability:Please further temper claims about generalizability, given the single-site VA setting and predominantly male sample. The limitations for non-VA, community-based, female, or more diverse populations should be clearly stated.

Minor comments

8.Please clarify the timing and funding source of financial incentives.

9.Ensure inclusion and exclusion criteria are both clearly listed.

10. Check the abstract for consistency with the adjusted results, especially age and race/ethnicity findings.

11. Because no multiple-comparison adjustment was performed, use exploratory language throughout.

12. Please use precise statistical wording, e.g., “All tests were two-sided, and p < 0.05 was considered statistically significant. P-values were not adjusted for multiple comparisons.”

13. In the response letter, include line numbers and avoid defensive wording such as “I believe.”

Overall, the study addresses an important recruitment question, but additional clarification of methods and reporting is needed to meet PLOS ONE standards for transparency and reproducibility.

**Reviewer #4:**  Table 2 (Crude analyses):

Please clarify whether Table 2 crude analyses represent (i) univariable logistic regression results, or (ii) LASSO-based variable selection/stability results across imputed datasets. If the latter is intended, this should be clearly relabelled and described in the statistical analyses section to avoid misinterpretation. If univariable logistic regression was performed, pooled estimates under Rubin’s Rules should be reported consistently for all variables.

**********

what does this mean?). If published, this will include your full peer review and any attached files.

If you choose “no”, your identity will remain anonymous but your review may still be made public.

**Do you want your identity to be public for this peer review?**  For information about this choice, including consent withdrawal, please see our Privacy Policy

Reviewer #2: No

Reviewer #4: No

**********

[NOTE: If reviewer comments were submitted as an attachment file, they will be attached to this email and accessible via the submission site. Please log into your account, locate the manuscript record, and check for the action link "View Attachments". If this link does not appear, there are no attachment files.]

To ensure your figures meet our technical requirements, please review our figure guidelines: https://journals.plos.org/plosone/s/figures

You may also use PLOS’s free figure tool, NAAS, to help you prepare publication quality figures: https://journals.plos.org/plosone/s/figures#loc-tools-for-figure-preparation.

NAAS will assess whether your figures meet our technical requirements by comparing each figure against our figure specifications.

# Author comment: Author response to Decision Letter 3

**DOI:** 10.1371/journal.pone.0345267.r006

21 Sep 2026

Reviewer #2

Major comments

1. Allocation concealment: The authors describe lack of blinding, but allocation concealment for the parent FIESTA trial remains unclear. Please briefly describe the randomization/allocation concealment procedure or cite the parent trial protocol/publication. If not relevant to this recruitment analysis, please state this explicitly.

Response: The randomization procedure is not relevant to this study as we are solely commenting on the recruitment process, added in as (Lines 100-101 in the tracked changes version).

2. Outcome definition: Please clearly define the binary outcome used in the logistic regression. Does “participation” mean consent, enrollment, randomization, or baseline visit attendance? Use the same term consistently throughout the manuscript.

Response: We have clarified that primary outcome was enrollment in the FIESTA trial among eligible patients (0 = not enrolled and 1 = enrolled) in the Methods. We also revised the Results and tables to use “enrollment rate” for the proportion of eligible patients enrolled and “odds of enrollment” when reporting logistic regression results (Methods, Lines 137-139; Results, Lines 214-215; Table 1, Line 226).

We have replaced the terminology of recruitment with enrollment consistently throughout the paper.

3. Missing data: The manuscript reports the percentage range of missing data, but not the missing-data pattern. Please describe whether missingness was monotone or non-monotone, whether variables were commonly missing together, and why MICE was considered appropriate.

Response: We have expanded the Statistical Analyses section to describe the pattern of missing data and clarify the rationale for using MICE. We clarified that MICE was used given the non-monotone pattern of missingness across multiple covariates and its ability to accommodate the continuous and categorical variables included in the analysis (Methods, Lines 163-166).

4. Model selection and confounding: The LASSO variable-selection procedure needs clearer description. Please specify candidate variables, the threshold for being “consistently retained” across imputed datasets, and how clinically important confounders were handled if not selected by LASSO.

Response: We have revised the Statistical Analyses section to clarify the LASSO variable-selection procedure, including the candidate predictors and how selection across the imputed datasets was evaluated. We also clarified that all prespecified clinically relevant demographic and smoking-related covariates were retained in the final multivariable model; therefore, no clinically important candidate confounders were excluded based on LASSO selection (Methods, Lines 168-177).

5. Model fit: VIF and fraction of missing information do not assess logistic regression model fit. Please report appropriate model performance/fit measures, such as C-statistic/AUC, calibration, Hosmer–Lemeshow test, or justify why these were not assessed.

Response: We have added the AUC as a measure of model discrimination and clarified its assessment in the Statistical Analyses section. The model demonstrated moderate discrimination across the imputed datasets (Methods, Lines 189-191; Results, Lines 219-221).

6. Ethics and data availability: Please ensure the ethics statement and data availability statement are consistent across the manuscript and submission system. The IRB approval should be stated definitively, including the approving IRB and approval number if available.

Response: We have included the protocol number in this study (Line 107) and ensured consistency across manuscript and submission system.

7. Generalizability: Please further temper claims about generalizability, given the single-site VA setting and predominantly male sample. The limitations for non-VA, community-based, female, or more diverse populations should be clearly stated.

Response: A line has been added further emphasizing the limited generalizability of our conclusions (Line 274-275).

Minor comments

8.Please clarify the timing and funding source of financial incentives.

Response: The timeline and source of funding for participants is stated in lines 104-105.

9.Ensure inclusion and exclusion criteria are both clearly listed.

Response: Inclusion and exclusion criteria are stated more plainly (Line 121-126).

10. Check the abstract for consistency with the adjusted results, especially age and race/ethnicity findings.

Response: The abstract is now consistent with the adjusted results. Older age was associated with lower enrollment and Hispanic race/ethnicity experienced greater enrollment as compared to White patients (Line 41-44).

11. Because no multiple-comparison adjustment was performed, use exploratory language throughout.

Response: We have revised the manuscript throughout to use more cautious, exploratory language when describing and interpreting the findings. (Abstract, Lines 36-46; Discussion, Lines 253, 283, 308-310).

12. Please use precise statistical wording, e.g., “All tests were two-sided, and p < 0.05 was considered statistically significant. P-values were not adjusted for multiple comparisons.”

Response: We have revised the Statistical Analyses section to the suggested wording (Methods, Lines 192-194).

13. In the response letter, include line numbers and avoid defensive wording such as “I believe.”

Response: We have avoided the use of defensive wording in our response letter and have provided line numbers when appropriate.

Reviewer #4: Table 2 (Crude analyses):

Please clarify whether Table 2 crude analyses represent (i) univariable logistic regression results, or (ii) LASSO-based variable selection/stability results across imputed datasets. If the latter is intended, this should be clearly relabeled and described in the statistical analyses section to avoid misinterpretation. If univariable logistic regression was performed, pooled estimates under Rubin’s Rules should be reported consistently for all variables.

Response: We have clarified that the crude estimates represent univariable logistic regression analyses rather than LASSO-based variable selection results. Crude estimates for all variables were pooled using Rubin’s Rules (Methods, Lines 187-189; Table 2).

Attachment Submitted filename: FIESTA manuscript Plos One Resonse to Reviewers - 8-31-2026.docx

# Peer review: Decision Letter 3

**DOI:** 10.1371/journal.pone.0345267.r007

**Authors:** Marc Potenza

12 Oct 2026

<p>An analysis of enrollment rates by admitting service for a secondary analysis of enrollment data from a randomized smoking-cessation trial (FIESTA)

PONE-D-25-20584R3

Dear Dr. Sherman,

We’re pleased to inform you that your manuscript has been judged scientifically suitable for publication and will be formally accepted for publication once it meets all outstanding technical requirements.

Within one week, you’ll receive an e-mail detailing the required amendments. When these have been addressed, you’ll receive a formal acceptance letter and your manuscript will be scheduled for publication.

An invoice will be generated when your article is formally accepted. Please note, if your institution has a publishing partnership with PLOS and your article meets the relevant criteria, all or part of your publication costs will be covered. Please make sure your user information is up-to-date by logging into Editorial Manager at Editorial Manager® and clicking the ‘Update My Information' link at the top of the page. For questions related to billing, please contact billing support.

If your institution or institutions have a press office, please notify them about your upcoming paper to help maximize its impact. If they’ll be preparing press materials, please inform our press team as soon as possible -- no later than 48 hours after receiving the formal acceptance. Your manuscript will remain under strict press embargo until 2 pm Eastern Time on the date of publication. For more information, please contact onepress@plos.org.

Kind regards,

Marc N Potenza

Academic Editor

PLOS One

Additional Editor Comments (optional):

Reviewers' comments:

Reviewer's Responses to Questions

**Comments to the Author**

Reviewer #2: All comments have been addressed

Reviewer #4: (No Response)

**********

2. Is the manuscript technically sound, and do the data support the conclusions?

Reviewer #2: No

Reviewer #4: (No Response)

**********

3. Has the statistical analysis been performed appropriately and rigorously?

Reviewer #2: Yes

Reviewer #4: (No Response)

**********

4. Have the authors made all data underlying the findings in their manuscript fully available?

The PLOS Data policy

Reviewer #2: Yes

Reviewer #4: (No Response)

**********

5. Is the manuscript presented in an intelligible fashion and written in standard English?

Reviewer #2: Yes

Reviewer #4: (No Response)

**********

Reviewer #2: Thank you for the revised manuscript. The paper has improved since the prior version, particularly in clarifying that this is a secondary, exploratory analysis of FIESTA enrollment data, defining the binary enrollment outcome, adding more detail on missing data, and reporting crude and adjusted logistic regression estimates. I also appreciate the added statement that p-values were not adjusted and should be interpreted as exploratory given the secondary nature of the analysis.

However, several issues remain before the manuscript is ready for publication.

1. Please revise the primary objective to be non-directional. The Introduction states the aim was to determine whether Medicine enrollment was higher than Psychiatry, but the results show higher enrollment in Psychiatry.

2. Clarify that allocation concealment in the parent trial did not affect this secondary analysis because enrollment was assessed before treatment assignment. Avoid saying randomization is “not relevant.”

3. Use enrollment terminology consistently. Table 1 still defines the outcome as “did not participate/participated”; revise to “not enrolled/enrolled.”

4. Please define “official enrollment” operationally, e.g., whether it means written informed consent, baseline completion, or randomization.

5. The missing-data pattern suggests possible informative missingness: education missingness was concentrated among non-enrolled patients, and cigarettes/day was missing only among non-enrolled patients. Please clarify whether missingness was related to non-completion of baseline assessment and whether the imputation model included enrollment status.

6. The role of LASSO remains unclear because all six prespecified variables were retained in the final model. Please state that LASSO was used as a supplementary stability check rather than the basis for final variable selection.

7. AUC assesses discrimination only. Please add a calibration assessment or explain why calibration/model fit was not evaluated.

8. Conclusions remain overstated. A single-site VA secondary analysis cannot establish that smoking-cessation programs broadly “effectively enroll” diverse populations or that interventions for patients with mental health diagnoses “would yield improved outcomes.”

9. Please avoid strong “significant” language given that p-values were unadjusted and exploratory.

10. Minor edits: correct “randomized control trial,” “V A,” “bio confirmed,” “FIRSTA,” and grammar in the model discrimination sentence.

Reviewer #4: (No Response)

**********

what does this mean?). If published, this will include your full peer review and any attached files.

If you choose “no”, your identity will remain anonymous but your review may still be made public.

**Do you want your identity to be public for this peer review?**  For information about this choice, including consent withdrawal, please see our Privacy Policy

Reviewer #2: **Yes:**  Wanjia He

Reviewer #4: No

**********

# Editor report: Acceptance letter

**DOI:** 10.1371/journal.pone.0345267.r008

**Authors:** Marc Potenza

PONE-D-25-20584R3

PLOS One

Dear Dr. Sherman,

I'm pleased to inform you that your manuscript has been deemed suitable for publication in PLOS One. Congratulations! Your manuscript is now being handed over to our production team.

At this stage, our production department will prepare your paper for publication. This includes ensuring the following:

* All references, tables, and figures are properly cited

* All relevant supporting information is included in the manuscript submission,

* There are no issues that prevent the paper from being properly typeset

You will receive further instructions from the production team, including instructions on how to review your proof when it is ready. Please keep in mind that we are working through a large volume of accepted articles, so please give us a few days to review your paper and let you know the next and final steps.

Lastly, if your institution or institutions have a press office, please let them know about your upcoming paper now to help maximize its impact. If they'll be preparing press materials, please inform our press team within the next 48 hours. Your manuscript will remain under strict press embargo until 2 pm Eastern Time on the date of publication. For more information, please contact onepress@plos.org.

You will receive an invoice from PLOS for your publication fee after your manuscript has reached the completed accept phase. If you receive an email requesting payment before acceptance or for any other service, this may be a phishing scheme. Learn how to identify phishing emails and protect your accounts at https://explore.plos.org/phishing.

If we can help with anything else, please email us at customercare@plos.org.

Thank you for submitting your work to PLOS One and supporting open access.

Kind regards,

PLOS One Editorial Office Staff

on behalf of

Dr. Marc N Potenza

Academic Editor

PLOS One
